# Diet and ideal food pyramid to prevent or support the treatment of diabetic retinopathy, age-related macular degeneration, and cataracts

**DOI:** 10.3389/fmed.2023.1168560

**Published:** 2023-05-30

**Authors:** Mariangela Rondanelli, Clara Gasparri, Antonella Riva, Giovanna Petrangolini, Gaetan Claude Barrile, Alessandro Cavioni, Claudia Razza, Alice Tartara, Simone Perna

**Affiliations:** ^1^Istituto di Ricovero e Cura a Carattere Scientifico (IRCCS) Mondino Foundation, Pavia, Italy; ^2^Unit of Human and Clinical Nutrition, Department of Public Health, Experimental and Forensic Medicine, University of Pavia, Pavia, Italy; ^3^Endocrinology and Nutrition Unit, Azienda di Servizi alla Persona “Istituto Santa Margherita”, University of Pavia, Pavia, Italy; ^4^R&D Department, Indena SpA, Milan, Italy; ^5^Department of Biology, College of Science, University of Bahrain, Zallaq, Bahrain

**Keywords:** diet, food pyramid, diabetic retinopathy, age-related macular degeneration, cataract, phytoextracts

## Abstract

Many eye diseases, such as diabetic retinopathy (DR), age-related macular degeneration (AMD), and cataracts are preventable and treatable with lifestyle. The objective of this review is to assess the most recent research on the ideal dietary approach to prevent or support the treatment of DR, AMD, and cataracts, as well as to construct a food pyramid that makes it simple for people who are at risk of developing these pathologies to decide what to eat. The food pyramid presented here proposes what should be consumed every day: 3 portions of low glycemic index (GI) grains (for fiber and zinc content), 5 portions (each portion: ≥200 g/day) of fruits and vegetables (spinach, broccoli, zucchini cooked, green leafy vegetables, orange, kiwi, grapefruit for folic acid, vitamin C, and lutein/zeaxanthin content, at least ≥42 μg/day, are to be preferred), extra virgin olive (EVO) oil (almost 20 mg/day for vitamin E and polyphenols content), nuts or oil seeds (20–30 g/day, for zinc content, at least ≥15.8 mg/day); weekly: fish (4 portions, for omega-3 content and eicosapentaenoic acid (EPA) + docosahexaenoic acid (DHA) 0.35–1.4 g/day), white meat (3 portions for vitamin B12 content), legumes (2 portions for vegetal proteins), eggs (2 portions for lutein/zeaxanthin content), light cheeses (2 portions for vitamin B6 content), and almost 3–4 times/week microgreen and spices (saffron and curcumin). At the top of the pyramid, there are two pennants: one green, which indicates the need for personalized supplementation (if daily requirements cannot be met through diet, omega-3, and L-methylfolate supplementation), and one red, which indicates that certain foods are prohibited (salt and sugar). Finally, 3–4 times per week, 30–40 min of aerobic and resistance exercises are required.

## 1. Introduction

The 2019 WHO World Report on Vision confirms that more than a billion people worldwide suffer from vision disorders, which can be prevented or treated to avoid blindness. Furthermore, the number of people suffering from partial or severe blindness is increasing alarmingly. Cataracts and refractive defects make up half of the cases of blindness or severe vision impairment; diabetic retinopathy (DR) is the major cause among persons of working age, whereas age-related macular degeneration (AMD) is the major cause in the elderly (1). While there is a surgical treatment for cataracts, there is still no cure for many eye conditions that cause blindness. Among these is AMD. This is why it is very important to study mechanisms that lead to disease and to slow down the progression through prevention. Diet and lifestyle are two of the most important thoroughly studied factors, but are still little known by patients. Both seem to significantly influence the onset of the disease and rate of progression. Many eye diseases are treatable and preventable, especially in the first phase in which they occur and lifestyle, understood as nutrition and physical activity (PA), plays an essential role. The growth of studies in the literature on the subject suggests that various eye diseases, including glaucoma, AMD, and DR are associated with lower levels of physical activity. Similarly, physical activity levels are lower in people with lower vision (2). The relationship between PA and three of the most common vision diseases has abundant evidence supporting a possible protective role of PA against vision loss. A very recent narrative review (3) analyzed evidence in the literature between dietary styles and common eye diseases: specifically, the authors conclude that there is enough evidence in the literature to suggest that the Mediterranean diet and the “Asian diet” are linked with a reduced incidence of AMD, whereas the Western diet is associated with a greater incidence. Moreover, there seems to be evidence of a positive correlation between the Western diet and the development of cataracts, while there are not enough data to identify a correct dietary style that prevents this pathology. The data currently available come mainly from observational studies and some randomized clinical studies related to nutritional epidemiology. Among these, the most important to-date remain the Age-Related Eye Disease Study (AREDS) and AREDS2 studies (4, 5).

### 1.1. Antioxidants

#### 1.1.1. Carotenoids

Only zeaxanthin and meso-zeaxanthin (a lutein metabolite formed in the macula through metabolic transformation) are present in significant amounts in the macula of human plasma (6). Together, these two carotenoids form the pigment of the macula, an essential component for maintaining vision at optimal levels, and this pigment can be used as a marker to assess the risk of AMD. Diet and supplements can alter the concentration of lutein and zeaxanthin, and thus their potential biological function. In 1997, the study by Hammon showed that the modification of one's diet can modify retinal receptor density: the addition of 60 mg of spinach (10.8 mg of lutein, 0.3 mg of zeaxanthin, and 5 mg of b-carotene) and/or 150 g of corn (0.4 mg of lutein and 0.3 of zeaxanthin) for 15 weeks to one's daily diet affected retinal receptor density (+19%) in 8 out of 12 subjects studied (7). In 2007, Schalch administered lutein (10 mg), zeaxanthin (10 mg), or a combination of the two (10+10 mg) to 126 male subjects for 1 year to assess their ability to influence macular pigment optical density (MPOD), by measuring monthly retinal parameters. It was found that the administration of one of the two components alone or the combined administration of both can improve MPOD up to 15% of the initial value. Furthermore, it was found that lutein tends to act mainly on the fovea, while zeaxanthin acts on the entire surface of the retina (8). Johnson (2008) investigated the effect of lutein (12 mg) and docosahexaenoic acid (DHA) (800 mg) supplementation for 4 months in preventing AMD in a sample of 49 women (aged 60–80 years): the subjects were randomized into four treatment groups (placebo, DHA, lutein, and DHA+lutein) and were evaluated before and after treatment for blood parameters and MPOD. The study showed that both lutein and DHA can significantly increase MPOD individually and in combination (*p* < 0.01) at 2 and 4 months after administration (9). Another feature of lutein is that it can be retained in the human retina for an extended period. In the study carried out by Landrum, two healthy subjects were supplemented with lutein esters equivalent to 30 mg of free lutein for 140 days, and during the intake period, it was possible to highlight a significant increase (+20–40% depending on the eye and the subject analyzed) of the MPOD, and this parameter continued to increase up to 50 days after the suspension of the supplementation and then progressively decreased (10). The articles by Eisenhauer and Perry report the content of lutein and zeaxanthin in foods; foods rich in lutein (lutein>900 mg/100 g) are in descending order of content: cooked spinach, cooked kale, cilantro, raw spinach, parsley, green leafy vegetables (lettuce and romaine), pistachios, zucchini cooked with skin, cooked asparagus. Foods rich in zeaxanthin (zeaxanthin>500 mg/100 g) are in descending order of content: scallions cooked in oil, oranges, raw egg yolk, and cooked egg yolk (11, 12).

The behavior of carotenoids in cooking has been investigated in the literature, but mainly for lutein, while studies on zeaxanthin are lacking. From a review by Palermo (13) regarding the effects of cooking on phytochemicals, several studies have analyzed lutein content in various vegetables before and after different types of cooking. Lutein tends to increase with steam cooking, probably due to the degradation of cellulose which allows for greater release and tends to be reduced with frying in proportion to the temperatures and surfaces exposed to cooking (cutting into smaller pieces tends to increase the surface in contact with the oil and therefore the loss of lutein). Evidence is conflicting for microwave cooking. A new frontier in the food sector is the use of “microgreens” or young seedlings (harvested 7–21 days after sowing) of various species of vegetables, wild plants, and aromatic herbs. These foods are richer in vitamins, micronutrients, and antioxidant compounds more than matured vegetables and plants. Xiao analyzed the content of lutein, zeaxanthin, tocopherol, beta carotene, and violaxanthin of these “young” vegetables (14): the microgreens richest in beta carotene (beta carotene:>10 mg/100 g fresh weight) are: cilantro, peppercress, red cabbage, and red sorrel, while the microgreens richest in lutein/zeaxanthin (lutein/zeaxanthin>8 mg/100 g fresh weight) include cilantro, garnet amaranth, and red cabbage. In consideration of these high contents of compounds useful for eye health, microgreens can be an excellent addition to a diet aimed at the prevention of eye diseases. Egg yolk is the finest non-vegetarian food source of lutein and zeaxanthin because eggs' high-fat content boosts the absorption of carotenoids (15), even though their level mostly depends on the hen's diet, which includes lutein and zeaxanthin in its esterified forms along with trace amounts of lycopene and β-carotene (16). In-depth knowledge of release into the circulation and before that of the absorption, transport, and accumulation of carotenoids in the eye is essential to evaluate their beneficial aspects. Carotenoids are generally lipophilic, however, lutein and zeaxanthin are more polar substances than hydrocarbon carotenoids like beta-carotene and lycopene because of the presence of the hydroxyl group. Lutein and zeaxanthin absorption from meals determines their bioavailability in ocular tissue (17), and intestinal absorption is in turn influenced by several factors: the type of the food matrix (natural food or supplement), the amount and type of fats consumed, which let carotenoids circulate, the potential existence of phospholipids, and the availability of dietary fiber. The characteristics of the food matrices have a significant impact on the bioavailability of carotenoids (18). Lutein, zeaxanthin, and beta-cryptoxanthin have been found to release almost completely from fruits (orange, kiwi, grapefruit, and sweet potato), but only 19–38% from green vegetables (spinach and broccoli) (19). Human tissues do not all contain the same amounts of lutein, with the macula having the highest concentration (20).

#### 1.1.2. Vitamin A

Unsaturated isoprenoid chain structure distinguishes the group of fat-soluble, vegetal, and animal-derived chemicals known as vitamin A and, in general, they are defined “retinoids.” All vitamin A types have the same physiological effects on an organism and a comparable structural makeup and they could be either from a natural or synthetic source. Unlike water-soluble vitamins, all of these substances are liposoluble and can easily accumulate in the body, particularly in the liver and adipose tissue (21). In this instance, 11-cis-retinol is the active vitamin A derivate; it is connected to the G-coupled protein receptor in the retina known as opsin. The complex is referred to as rhodopsin, and it is the essential pigment for seeing in the dark (22). Vitamin A deficiency, common in the presence of generalized malnutrition, is associated with night blindness, conjunctival xerosis, and corneal ulceration, particularly with concomitant measles infection (23, 24). Two recent reviews have shown significant effects of vitamin A in preventing ocular diseases such as cataracts: data from the meta-analysis presented by Wang A et al. showed that ingesting enough vitamin A decreased the risk of cataracts by 17% (95% CI, 0.757–0.913) (25) and the review of Jiang H et al. showed a significant reduced risk of cataract by the consumption of carotenoids [relative risk (RR), 0.81; 95% CI, 0.71–0.92] (26). Although data from the National Health and Nutrition Examination Survey (NHANES I) initially showed a protective effect of a diet based on the high amount of fruit and vegetables rich in vitamin A on developing AMD (27), the following epidemiological studies did not found any significative evidence on the association between dietary intake of vitamin A and reduced risk of AMD (28), so further investigations are needed.

In light of this background, the objective of this review is to assess the most recent information regarding the ideal dietary approach to prevent or support the treatment of DR, AMD, and cataracts, and to construct a food pyramid that enables subjects who are at risk of developing these pathologies or subjects who have these pathologies to easily figure out what to eat.

**Figure 1:**
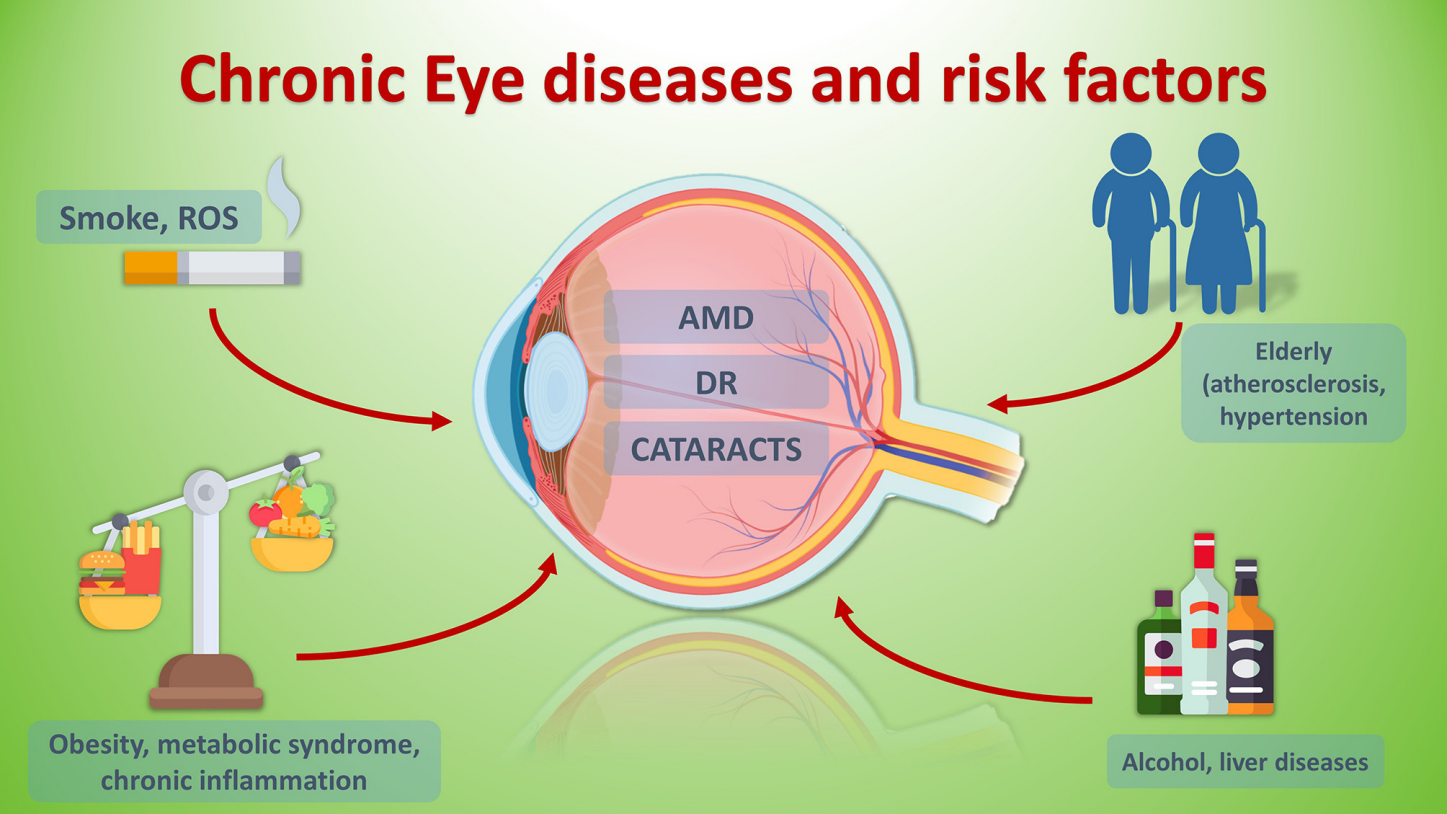
Chronic eye disease and risk factors.

Figure 1 summarizes the main risk factors common to the three eye diseases discussed in the review.

## 2. Methods

The procedures used to carry out this narrative review are as follows (29): (1) Three clinical nutrition-trained operators compose the working group (one acting as a methodological operator and two participating as clinical operators); (2) Formulation of the revision question based on the abstract's points: “the most recent information on the optimal dietary approach to prevent or support the treatment of DR, AMD, and cataracts”; (3) Identification of pertinent studies: The following research method was planned on PubMed [Public MEDLINE, operated by the National Center for Biotechnology Information (NCBI) of the National Library of Medicine of Bethesda (Bethesda, MD, USA)]: (a) the definition of the keywords (DR, foods, nutrients, and diet), which can be used singly or in combination, (b) the use of the Boolean operator, which enables the establishment of logical relationships between concepts, (c) advanced search as a research modality, (d) Limitations: human subjects; English; articles published within the last 30 years; and (e) manual search by senior researchers skilled in clinical nutrition through the revision of reviews and particular patient dietary therapy publications published in journals qualified in the Index Medicus; (4) analysis and presentation of outcomes: the data extrapolated from the “revised studies” were allocated in tables; in particular, for each study, the authors, year of publication, and study characteristics were reported; (5) A narrative review of the reports was used to carry out the analysis. Each section's introduction includes a list of the studies that were considered as well as the type of study and keywords. We reviewed studies of any design that took account of the importance of diet, foods, nutrients, and dietary patterns (DPs) to prevent or support the treatment of DR, AMD, and cataracts.

**Figure 2:**
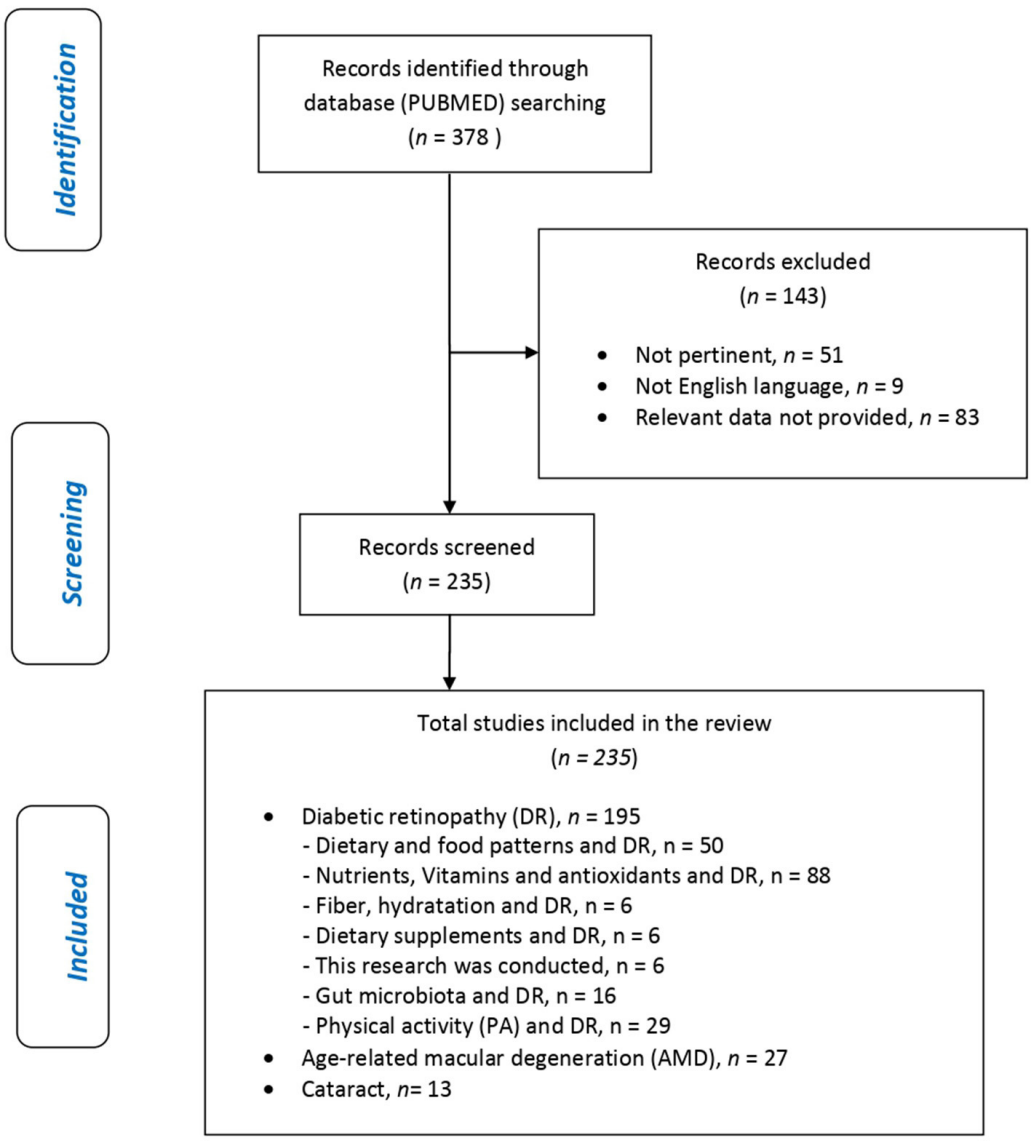
Flow chart.

**Figure 3:**
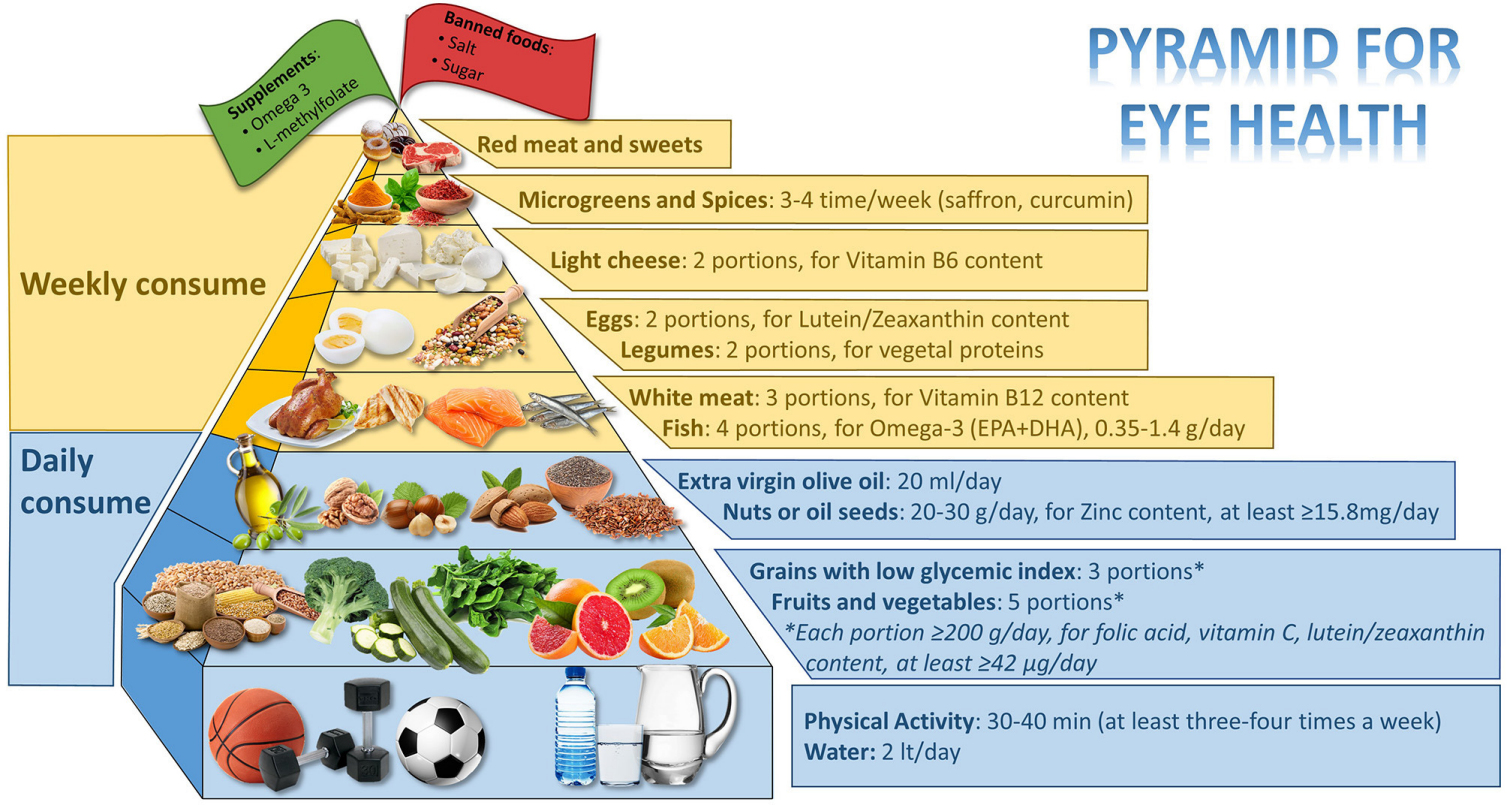
Food pyramid for eye health.

Figure 2 shows the eligible studies and Figure 3 represents proper nutrition and lifestyle to prevent or support the treatment of DR, AMD, and cataracts, specifying the quality and amount of food needed to provide ideal dietary management and to construct a food pyramid.

## 3. Results

### 3.1. DR

#### 3.1.1. Dietary and food patterns

The following keywords served as the basis for this research: “diet” OR “Mediterranean diet” OR “natural food “OR “Fruits and vegetables” OR “nuts” OR “saffron” OR “curcumin” OR “Tea and coffee” AND “diabetic retinopathy” OR “eye diseases” OR “diabetes.” Thirty-one articles were consulted, including five narrative reviews, four systematic reviews, one systematic review & meta-analysis, a review of *in vitro* studies, 11 clinical trials (two *post hoc* analyses of randomized trials, one cross-sectional study, one retrospective study, two cohort studies, one randomized controlled trial, two case–control studies, and one prospective trial), six *in vitro* studies, and three studies on animal models.

Table 1 shows the studies that evaluated the relationship between DPs and food and diabetic retinopathy with their strength of evidence.

**Table 1:** Dietary and food patterns and DR.

**References**	**Study design**	**Study period**	**Endpoint**	**Subjects (age, sex, number…)**	**Method**	**Results**	**Strength of evidence**
Diaz-Lopez et al. (30)	*Post-hoc* analysis of a randomized trial	6 years (from 2003 to 2009)	To determine the effect of the three dietary interventions on the incidence of diabetes complications.	Type 2 diabetes participating in the “PREvencion con DIeta MEDiterranea (PREDIMED)” randomized clinical trial, who were free of microvascular complications at enrolment (*n* = 3,614, aged 55–80 years)	Patients were randomly assigned to one of three dietary interventions: MedDiet supplemented with extravirgin olive oil (MedDiet+EVOO), MedDiet supplemented with mixed nuts (MedDiet+Nuts), or a low-fat control diet. Two independent outcomes were considered: new onset of diabetic retinopathy and nephropathy. Hazard ratios (HRs) were calculated using multivariable-adjusted Cox regression.	Compared with the control diet, multivariable-adjusted HRs for diabetic retinopathy were 0.56 (95% CI 0.32–0.97) for the MedDiet+EVOO and 0.63 (0.35–1.11) for the MedDiet+Nuts. No between-group differences were found for nephropathy.	Moderate
Salas-Salvadó et al. (31)	*Post-hoc* analysis of a randomized trial	8 years (2003 to 2011)	To assess the efficacy of Mediterranean diets for the primary prevention of diabetes in the Prevención con Dieta Mediterránea trial.	7447 partecipants at high cardiovascular risk, aged between 55 and 80 years (57% were female)	Participants were randomly assigned and stratified by site, sex, and age but not diabetes status to receive 1 of 3 diets: Mediterranean diet supplemented with extra-virgin olive oil (EVOO), Mediterranean diet supplemented with nuts, or a control diet (advice on a low-fat diet). No intervention to increase physical activity or lose weight was included.	A Mediterranean diet enriched with EVOO but without energy restrictions reduced diabetes risk among persons with high cardiovascular risk.	Moderate
Sala-Vila et al. (32)	Prospective randomized trial	6 years (From 2003 to 2009)	To determine whether LCω3PUFA intake relates to a decreased incidence of sight-threatening DR in individuals with type 2 diabetes older than 55 years	3,614 individuals aged 55 to 80 years with a previous diagnosis of type 2 diabetes, participating in the “PREvencion con DIeta MEDiterranea (PREDIMED)” randomized clinical trial, considering only subjects with type 2 diabetes at baseline.	Dietary intake was assessed at baseline and yearly during follow-up up by using a 137-item semiquantitative food-frequency questionnaire validated for the PREDIMED study. Information on seafood products was collected in 8 items of the questionnaire (uncanned oily fish; lean fish; smoked/salted fish; mollusks; shrimp, prawn, and crayfish; octopus, baby squid and squid; oily fish canned in oil; and oily fish canned in salted water).	In middle-aged and older individuals with type 2 diabetes, intake of at least 500 mg/d of dietary LCω3PUFA, easily achievable with 2 weekly servings of oily fish (as recommended in the Mediterranean diet), is associated with a decreased risk of sight-threatening DR.	Moderate
El Bilbeisi et al. (33)	Cross sectional study	2 years (from 2015 to 2016)	To identify major dietary patterns among DM2 pt and its association with diabetes complications	1,200 T2DM patients, selected by a cluster random sampling method, aged 20–64 years receiving care in the primary healthcare centers (PHCs) in Gaza Strip, Palestine, (59.8% females, 40.2% males)	Data about dietary patterns were collected by an expert nutritionist, using a validated semi-quantitative food frequency questionnaire (FFQ). Dietary patterns were obtained using factor analysis after the classification of food items into 25 groups.	The Asian-like dietary pattern (high intake of whole grains, potatoes, beans, legumes, vegetables, tomatoes and fruit) is associated with a lower prevalence of diabetes complications among DM2 patients rather than sweet-soft drinks-snacks pattern	Low
Tanaka et al. (34)	Cohort	8 years (from 1995 to 2003)	To establish the effect of fruit & vegetables (Vitamin C, Vitamin E, Carotenoids, fiber) intake on DR incidence	This study is part of the Japan Diabetes Complications Study, an open-labeled randomized trial originally designed evaluate the efficacy of a long-term therapeutic intervention focused on lifestyle education. It included 978 patients aged 40–70 years.	Baseline dietary intake was assessed by a food frequency questionnaire based on food groups and 24-h dietary records. Primary outcome was incident diabetic retinopathy determined using international severity scales.	Adequate fruit consumption of 173.2 g per day was associated with a 50% reduced risk of DR incidence, compared with consumption of 53.2 g of fruit per day or less	Low
Bazzano et al. (35)	Retrospective	4 years (from 1971 to 1975)	To examine the association of fruit and vegetable intake with the risk of cardiovascular disease	9608 adults aged 25–74 years participating in the first National Health and Nutrition Examination Survey Epidemiologic Follow-up Study and free of cardiovascular disease at the time of their baseline examination.	Fruit and vegetable intake at baseline was measured with a food-frequency questionnaire.	The frequency of fruit and vegetable intake is inversely associated with stroke incidence and mortality, ischemic heart disease mortality, cardiovascular disease mortality, and all-cause mortality in the general US population. fruit and vegetables should be consumed in each meal in adequate quantities equal to at least 400 g per day. Each additional serving of fruit and vegetables reduces the risk of cardiovascular events by 4% and stroke by 5%	Low
Sala-Vila et al. (36)	Randomized controlled trial	2 years	To examinate the cognitive effects of a 2-years walnut intervention in cognitively healthy elders	636 participants (63–79 yeras old, 68% women) of the Walnuts And Healthy Aging (WAHA) study (Barcelona, Spain; Loma Linda, CA)	The participants were randomly allocated to a diet enriched with walnuts at ~15% energy (30–60 g/d) or a control diet (abstention from walnuts). a comprehensive neurocognitive test battery was administered at baseline and 2 years.	Walnut supplementation for 2 y had no effect on cognition in healthy elders. However, brain fMRI and *post-hoc* analyses by site suggest that walnuts might delay cognitive decline in subgroups at higher risk.	Moderate
Nunes et al. (37)	Case-control study	/	To characterize the association of lifestyle and nutritional risk profiles with age-related macular degeneration (AMD) in two subpopulations with differing AMD prevalence.	1,992 participants included 768 patients with AMD and 1,224 age- and sex-matched participants without AMD	Enrolled participants completed a validated lifestyle and food frequency questionnaire. A score to measure adherence to the Mediterranean diet (mediSCORE; Range, 0–9) was constructed from individual food intakes, which were further analyzed by conversion to nutrient consumption	High adherence to a Mediterranean diet and regular physical activity seem to be protective factors for AMD in a Portuguese population. The effect of the diet is likely driven by the increased consumption of vegetables, fruits, and nut.	Low
Ma et al. (38)	Case-control study	1 year (2013)	To determine the association between regular Chinese green tea consumption and the risk of DR in diabetic patients in China.	200 patients: 100 DR patients and 100 age-sex-matched diabetic controls without retinopathy, including 68 men and 132 women aged 35–85 years	DR was defined from retinal photographs and detailed information on Chinese green tea consumption of the participants was collected through a face-to-face interview.	Diabetic patients who had regularly drunk Chinese green tea every week for at least 1 year in their lives had a DR risk reduction of about 50% compared with those who had not.	Low
Hjellvik et al. (39)	Cohort	4 years (from 2004 to 2008)	To study the association between consumption of filtered boiled coffee consumption and incident of type 2 diabetes.	3,62,045 Norwegians (1,71,414 Men and 1,90,631 Women) aged 40–45 years old at the time of health survey administration.	Information on self-reported coffee consumption was available from health surveys conducted from 1985 to 1999 in Norway. Type 2 diabetes incidences were extimated from redeemed prescriptions of oral antidiabetic drugs in the period 1 January 2004 to 1 January 2008 from the Norwegian Prescription Database.	A moderate inverse association was found between consumption of both boiled and other types of coffee at the age of 40–45 years and the risk of being prescribed oral antidiabetic drugs 5–20 years later.	Low
Tuomilehto et al. (40)	Prospective study	mean follow-up of 12 years	To determine the relationship between coffee consumption and the incidence of type 2 DM among Finnish individuals, who have the highest coffee consumption in the world.	6,974 Finnish men and 7,655 women aged 35 m to 64 years without history of stroke, coronary heart disease, or DM at baseline	Information on self-reported coffee consumption was obtained from combined surveys conducted in 1982, 1987, and 1992. Mean follow-up of 12 years. Main Outcome Measures Hazard ratios for the incidence of type 2DM were estimated for different levels of daily coffee consumption	Coffee drinking has a graded inverse association with the risk of type 2 DM. In both sexes combined, the multivariate-adjusted inverse association was significant (P for trend 0.001) and persisted when stratified by younger and older than 50 years; smokers and never smokers; healthy weight, overweight, and obese participants; alcohol drinker and non-drinker; and participants drinking filtered and non-filtered coffee.	Low

#### 3.1.2. Nutrients, vitamins, and antioxidants and DR

This research was conducted based on the keywords: “nutrients” OR “Vitamins” OR “antioxidants” OR “Vitamin A and carotenoids “OR “vitamin E” OR “vitamin D and 25-hydroxyvitamin D” OR “Polyphenols” OR “vitamin C” OR “B vitamins” OR “Fatty acids” OR “zinc” AND “diabetic retinopathy” OR “eye diseases” OR “diabetes.” Forty-one studies have been referenced, including nine narrative reviews, one systematic review, 19 clinical trials (two cross-sectional studies, five retrospective studies, three cohort studies, five randomized controlled trials, two case–control studies, one prospective study, and one longitudinal study), seven *in vitro* studies, three animal model studies, one book, and one Health Professional Fact Sheet.

Table 2 shows the studies that evaluated the relationship between Nutrients, Vitamins, and antioxidants and diabetic retinopathy with their strength of evidence.

**Table 2:** Vitamins and antioxidants and DR.

**References**	**Study design**	**Study period**	**Endpoint**	**Subjects (age, sex, number…)**	**Method**	**Results**	**Strength of evidence**
Brazionis et al. (41)	Cross-sectional study	/	To evaluate the relationship between plasma carotenoids and diabetic retinopathy.	111 individuals with type 2 diabetes aged 44–77 years	Data for clinical and demographic variables and risk factors for diabetic retinopathy were obtained from 24 h urine and fasting blood samples, and an interviewer-assisted lifestyle questionnaire.	The study suggests synergies between carotenoids and DR, regardless of established risk factors. The results indicate that dietary modulation of retinopathy risk may be possible by increasing intakes of lutein- and lycopene-rich foods (higher plasma levels of lutein and zeaxanthin were associated with a lower risk of DR).	Low
Garcia-Medina et al. (42)	Randomized clinical trial	5 years	To evaluate the effect of antioxidant supplementation on DR over a 5-year follow-up period.	105 type 2 diabetic patients with nonproliferative DR, aged 41–68 years (53 males, 52 femakes).	A complete ophthalmic checkup and a plasma determination of oxidative [malonyldialdehyde (MDA)] and antioxidant parameters [total antioxidant status (TAS)] were obtained as the baseline. Patients were randomly assigned to the oral antioxidant supplementation group at nutritional doses (OASG) (*n* = 62) or to the absence of supplementation group (ASG) (*n* = 43). The best-corrected visual acuity, DR score, MDA, and TAS values were compared at the beginning and after 5 years (the end of the follow-up).	Best-corrected visual acuity did not change during the follow-up, irrespective of supplementation. However, the retinopathy stage showed a retardation of progression in the subgroup with supplementation, but worsened in the subgroup with no antioxidant supplementation. Furthermore, the antioxidant supplementation group maintained its antioxidant plasma status levels, which was related to decreased oxidative plasma activity.	Moderate
Zhang et al. (43)	Randomized, double-blind, placebo-controlled trial	9 months	To determine whether supplementation with lutein improved visual function in patients with non-proliferative diabetic retinopathy.	31 patients (8 females, 23 males) aged 40–85 years with type 2 diabetes and NPDR	The participants were assigned randomly to 10 mg/d of lutein or identical placebo for 36 weeks. Visual performance indices, including visual acuity (VA), contrast sensitivity (CS) and glare sensitivity (GS) at four different spatial frequencies, were measured at baseline, week 18 and 36.	In patients with NPDR, supplementation with lutein resulted in potential improvements in CS at low spatial frequency. Further studies are required to determine the possibility that such intervention could be used as an adjunct therapy to prevent vision loss in diabetic patients.	High
Moschos et al. (44)	Retrospective study	2 years	To investigate the effects of a carotenoid supplementation on retinal thickness and macular function of patients with diabetes using optical coherence tomography (OCT) and multifocal electroretinography (mfERG).	120 eyes of 60 patients age between 40 and 60 years with non-insulin dependent type 2 diabetes mellitus without diabetic retinopathy	Patients underwent OCT and mfERG and took vitamin supplements for a period of 2 years. Patients received a carotenoid supplement containing lutein (10 mg), zeaxanthin (2 mg) and meso-zeaxanthin (10 mg) once a day for 2 years. The thickness of the fovea was evaluated using OCT and the macular function was tested by mfERG.	OCT showed an increase in the central foveal thickness and mfERG revealed increased retinal response density within the central 13° surrounding the fovea (rings 1 to 3) at 2 years after the onset of carotenoids supplement intake. The use of carotenoid supplements may be of benefit for improving visual function of type 2 diabetes patients.	Low
Millen et al. (45)	Cohort study	From 1987 to 1998	To study the association between prevalent DR and intake of vitamins C and E in participants of the Atherosclerosis Risk in Communities Study.	A total of 1353 subjects, aged 45–64 years, with type 2 diabetes diagnosed between 1993 and 1995 or before were included. The subjects were extracted from the Atherosclerosis Risk in Communities Study (ARIC Study), who is a prospective study designed to investigate the etiology of atherosclerosis.	Participants were recruited to return 3 additional times after visit 1: visit 2 (1990–1992), visit 3 (1993–1995), and visit 4 (1996–1998). Dietary data were collected from participants at visits 1 and 3. Eye photographs of the participants were taken only at visit 3. Nutrient intake was assessed with a food-frequency and supplement questionnaire administered between 1987–1989 and 1993–1995. Prevalent retinopathy (*n* = 224) was determined in 1993–1995 from graded fundus photographs.	No association of DR with intake of vitamin C or E from food alone or from food and supplements combined was observed. A decreased odds of DR was found among users (reported use > or =3 y before 1993–1995) of vitamin C or E supplements or multisupplements compared with reported use of no supplements: 0.5 (0.3, 0.8), 0.5 (0.2, 0.8), and 0.4 (0.2, 0.9), respectively. Therefore, supplement use may reflect non-dietary factors or a possible benefit of supplementation.	Low
Mayer-Davis et al. (46)	Longitudinal study	/	to examine the relation between dietary and supplement intakes of vitamins C, E, and beta-carotene and the risk of DR.	A total of 387 participants with type 2 diabetes, from the San Luis Valley Diabetes Study, including non-Hispanic white and Hispanic adults in southern Colorado.	Ordinal logistic regression analysis was used, taking advantage of multiple clinic visits by individual participants and observations from both eyes, to assess the risk for increased DR severity over time as a function of changes in intake of vitamin C, vitamin E, and beta-carotene. Inteake of vitamina C, E and beta-carotene was obtained from a 24-h dietary recall (including vitamin supplement use). Six categories of intake for each nutrient (first to fourth quintiles and ninth and tenth deciles) were considered to ascertain any potential threshold effect. Analyses accounted for age, duration of diabetes, insulin use, ethnicity, glycated hemoglobin, hypertension, gender, and caloric intake.	No protective effect was observed between antioxidant nutrients and DR. Depending on insulin use, there appeared to be a potential for deleterious effects of nutrient antioxidants	Low
Millen et al. (47)	Cohort study	8 years (1987–1995)	To examine the association between vitamin D status and prevalent DR in participants with diabetes from a population-based cohort.	1339 participants (710 females and 629 males, aged 45–65 years) were included from the ARIC Study, recruited participants from different areas of USA. This study sample consists of Caucasian and African American participants with T2DM.	The study steps include 3 visit: at visit 1 (1987–1989) dietary intake of vitamin D was assessed, at visit 2 (1989–1992) the participants had serum 25-hydroxyvitamin (25[OH]D) concentrations assessed and at visit 3 (1993–1995) and non-mydriatic retinal photographs were taken. Logistic regression was used to estimate odds ratios (ORs) and 95 % confidence intervals (CIs) for diabetic retinopathy by categories of season-adjusted 25(OH)D (<30 [referent], 30– <50, 50– <75 and ≥75 nmol/L), by quartile of vitamin D intake (IU/day), and use of vitamin D or fish oil supplements (yes/no). P for trend was estimated using continuous 25(OH)D or vitamin D intake. ORs were adjusted for race, and duration of diabetes. A further adjustement was for HBA1c and hypertension to examine if 25(OH)D influenced diabetic retinopathy via its effects on either glycemic control or blood pressure.	ORs (95 % CIs) for retinopathy, adjusted for race and duration, were 0.77 (0.45–1.32), 0.64 (0.37–1.10), and 0.39 (0.20–0.75), p for trend = 0.001, for participants with 25(OH)D of 30– <50, 50– <75, and ≥75 nmol/L, respectively. No statistically significant association was observed between vitamin D intake from foods or supplements and retinopathy. Therefore, 25(OH)D concentrations ≥75 nmol/L were associated with lower odds of any retinopathy assessed 3 years later. The aauthors suggest this may be due in part to vitamin D's influence on blood glucose control.	Low
Long et al. (48)	Retrospective, population-based, cross-sectional study	3 years (2005–2008)	To evaluate the association between vitamin D deficiency and retinopathy severity in diabetic patients with poorly or well-controlled glycaemia.	The National Health and Nutrition Examination Survey (NHANES) 2005–2008 data were used for the study. The population included 842 adults (52.8% women) with mean age of 61.2 years.	Outcomes assessed included retinopathy severity, HbA1c levels, socioeconomic, behavioral, and biological factors. Univariate and multivariate regression analysis was used to evaluate association of different parameters with retinopathy severity. The interaction among HbA1c control, vitamin D deficiency, and retinopathy severity were also explored.	Multivariate ordinal regression analysis found being male (odds ratio (OR): 1.602, *P* = 0.001), increased duration of diabetes (OR: 1.072, *P* = 3.77E – 7) and poorly controlled HbA1c (OR: 3.522, *P* = 2.00E – 5) were associated with greater retinopathy severity. The association between vitamin D deficiency and DR severity only found in diabetic patients with well-controlled glycaemia.	Low
Mahoney et al. (49)	Retrospective, population-based, cross-sectional study	3 years (2003–2006)	To determine the relationship between dietary flavonoid-rich fruit and vegetable consumption on diabetes-related biomarkers (e.g., HgbA1c) and DR.	381 participants with diabetes from the NHANES study 2003–2006, mean age 61.4 years, 53.8% female.	Blood samples were taken to measure C-reactive protein (CRP), HgbA1C, and fasting glucose and insulin. Diabetic retinopathy was assessed from a retinal imaging exam. A high-flavonoid fruit and vegetable consumption (HFVC) index variable was created from a food frequency questionnaire (FFQ).	Greater HFVC was associated (p b 0.05) with lower levels of CRP (β = – 0.005), HgbA1C (β = – 0.005) and glucose (β = −0.59), with greater HFVC reducing the odds of having DR by 30%. Therefore, adults with diabetes consuming more flavonoid-rich fruits and vegetables had lower degrees of inflammation, better glycemic control, and reduced odds of DR.	Low
Park et al. (50)	Case-control prospective study	/	To determine whether vitreous level of vitamin C is compromised in patients with PDR and to investigate the association of diabetic macular ischemia and vitamin C.	40 patients (13 males, 27 females) who underwent pars plana vitrectomy for the treatment of PDR (PDR group, *n* = 20) and non-diabetic patients with idiopathic epiretinal membrane (control group, *n* = 20). PDR patients (60.4 ± 2.1 y) were younger than non-diabetic control patients (67.4 ± 1.2 y).	Serum, aqueous humor, and the vitreous were collected for the analysis of vitamin C level by HPLC. Diabetic macular ischemia (DMI) in PDR group was evaluated with fluorescein angiography (FA).	Vitreous level of vitamin C in PDR patients showed a 10 fold decrease, which was associated with the degree of macular ischemia. This suggests that vitreous vitamin C depletion may cause macula ischemia in PDR patients.	Low
Gurreri et al. (51)	Retrospective study	/	to demonstrate that statins and vitamin C (alone or in combination with statins) as complementary therapy could have an impact on the non-proliferative NPDR complication rate.	479 patients with NPDR	Statins and vitamin C intake were analyzed, along with the rate of diabetic macular edema (DME), vitreous hemorrhage (VH), circinate maculopathy (CM), and proliferative DR (PDR).	Statins, alone or with vitamin C, appear to reduce the complication rate of NPDR.	Low
Thosar et al. (52)	Randomized Controlled Trial	/	To test the hypothesis that antioxidant Vitamin C prevents the impairment of endothelial function during prolonged sitting.	Eleven men (24.2 ± 4.4 yrs)	Participants are randomized in 2 groups: the sitting without vitamin C (SIT) and the sitting with vitamin C (VIT). Participants were seated for 3 h without moving their legs. Additionally, in the VIT trial, participants ingested 2 vitamin C tablets (1 g and 500 mg) at 30 min and 1 h 30 min, respectively. Superficial femoral artery (SFA) flow-mediated dilation (FMD) was measured hourly for 3 h.	Three hours of sitting resulted in impaired SFA FMD. Antioxidant Vitamin C prevented the decline in SFA FMD, suggesting that oxidative stress may contribute to the impairment in endothelial function during sitting.	Moderate
Memisogullari et al. (53)	Case-control study	/	To assess the relationship between serum homocysteine, a potential parameter for atherosclerosis, and the ocular blood flow velocity and the resistivity index in highway toll collectors.	22 toll collectors (mean age 39,7 ±7,1) and 24 control subjects (mean age 38 ±6,7). All subjects are male.	The peak systolic and end diastolic flow velocities and the resistivity index were measured in 22 toll collectors and 24 control subjects by color Doppler ultrasonography. The resistivity index, which is an indirect measure of the atherosclerotic process, was calculated: resistivity index = (peak systolic velocity – end diastolic velocity)/peak systolic velocity. Serum homocysteine levels were determined by fluorometric high-performance liquid chromatography	There were significant correlations between the serum homocysteine level and ophthalmic artery resistivity index in both highway toll collectors (*p* < 0.001) and controls (*p* < 0.005). Exposure to exhaust particles might increase the serum homocysteine level, which in turn could lead to the decreased ocular blood flow and the increased resistivity index.	Low
Horikawa et al. (54)	Case-control study	/	To investigate the relationship between vitamin B6 intake and the incidence of diabetic retinopathy in Japanese patients with type 2 diabetes.	978 particiapnts from an examination of a nationwide cohort of patients with type 2 diabetes aged 40–70 years with HbA1c ≥ 48 mmol/mol, 47% female.	Cox regression analyses estimated hazard ratios (HRs) for retinopathy according to vitamin B6 intake adjusted for age, gender, body mass index, HbA1c, smoking, energy intake, and other confounders.	HRs for diabetic retinopathy in the 2nd, 3rd, and 4th quartile groups of vitamin B6 intake compared with the 1st quartile group were 1.17 (95% confidence interval 0.81–1.69, *p* = 0.403), 0.88 (0.58–1.34, *p* = 0.550), and 0.50 (0.30–0.85, *p* = 0.010), respectively. Findings suggested that high vitamin B6 intake was associated with a lower incidence of DR in Japanese with type 2 diabetes.	Low
Gopinath et al. (55)	Cohort study	/	To investigate associations between intakes and serum concentrations of folate and vitamin B-12 or serum tHcy and 10-y age-related macular degeneration (AMD) incidence.	1,390 participants, aged 59–78 years from the Blue Mountains Eye Study (BMES, a population-based cohort study of common eye diseases and other health outcomes in a suburban Australian population located west of Sydney. Steps of data collection: Baseline (1992–1994, BMES-1), after 5 y (1997–1999; BMES-2), 10 y (2002–2004; BMES-3), and 15 y (2007–2009; BMES-4).	Serum folate, vitamin B-12, and tHcy were determined from blood samples drawn in 1997–1999 from cohort members aged ≥55 y. AMD was assessed in 1,760 survivors from retinal photographs taken in 2002–2004 and 2007–2009. Total intakes of folate and vitamin B-12 were assessed by using a food-frequency questionnaire.	Elevated serum tHcy and folate and vitamin B-12 deficiencies predicted increased risk of incident AMD, which suggests a potential role for vitamin B-12 and folate in reducing AMD risk.	Low
Sasaki et al. (56)	Cross-sectional study	/	To assess the associations between dietary intake of polyunsaturated fatty acids (PUFAs) and DR.	379 patients (median age: 66.0 years, 66% male) with diabetes attending a diabetes eye clinic	Daily fatty acid intake was assessed by using a validated Food Frequency Questionnaire and adjusted for energy intake. Diabetic retinopathy was graded from fundus photographs as no DR, non-proliferative DR, or proliferative DR. Patients were categorized as “well-controlled diabetes” (*n* = 123) and “poorly controlled diabetes” (*n* = 256), defined as glycated hemoglobin (HbA1c) level <7.0% or ≥ 7.0%, respectively.	Increasing PUFA intake was associated with a reduced likelihood of the presence and severity of DR in well-controlled diabetes, whereas increasing saturated fatty acid intake was associated with an increased likelihood of the presence and severity of DR.	Low
Ansar et al. (57)	Randomized Controlled Trial	8 weeks	To examine the effects of alpha-lipoic acid (ALA) treatment over a period of 2 months on fasting blood glucose (FBG), insulin resistance (IR), and glutathione peroxidase (GH-Px) activity in type 2 diabetes (T2DM) patients.	57 type 2 DM patients (*n* = 57)	Patients were divided into 2 groups to receive either ALA (300 mg daily) or placebo by systematic randomization, and were followed-up for 8 weeks. After an overnight fasting and 2 h after breakfast, patients' blood samples were drawn and tested for FBG, 2 h PPG, serum insulin level, and GH-Px activity.	The study showed a significant decrease in FBG and PPG levels, IR-Homeostasis Model Assessment (IR-HOMA index) and GH-Px level in the ALA group. The comparison of differences between FBG and IR at the beginning and at the end of study in the ALA treated group and the placebo group were also significant. Therefore, this study supports the use of ALA as an antioxidant in the care of diabetic patients.	Moderate
Xiang et al. (58)	Randomized controlled trial	/	To examine endothelial dysfunction (ED) in the fasting state and after a glucose challenge as well as after administration of an antioxidant agent.	The study subjects included 42 with Impaired glucose tolerance (IGT) and 26 healthy individuals (control group).	The IGT patients were randomly divided into two groups, 21 in each group (the alpha-lipoic acid group and the placebo group). In the alpha-lipoic acid group, 300 mg of alpha-lipoic acid was administrated before an oral glucose tolerance test (OGTT); in the placebo group, 250 ml of 0.9% sodium chloride was administrated before the OGTT. In addition, 250 ml of 0.9% sodium chloride was also administrated to the control subjects before the OGTT (control group), and then vascular function was examined in the fasting state and repeated 1 and 2 h after the glucose load. High-resolution ultrasound was used to measure flow-mediated endothelium-dependent arterial dilation (FMD) and glyceryltrinitrate (GTN)-induced endothelium-independent arterial dilation.	In subjects with IGT, FMD was impaired both in the fasting state and after a glucose challenge, probably through increased production of oxygen-derived free radicals. The Endothelial dysfunction observed after a glucose challenge is related to the extent of hyperglycaemia and thiobarbituric acid reactive substances, and an antioxidant agent can improve the impairment of endothelial function induced by acute hyperglycaemia.	Moderate
Luo et al. (59)	Retrospective	1 years	To analyze the relationship between zinc level and each diabetic microvascular complication and identify the features related to low serum zinc level.	412 hospitalized T2D patients (233 males, 179 females, aged 41–71 years) In Department of Endocrinology, Peking University People's Hospital, Beijing, China, from May 30, 2013 to March 31, 2014.	The authors initially compared the serum zinc levels between patients with specific microvascular complications and those without. They then analyzed the association between zinc level and each microvascular complication. Furthermore, they identified the unique features of patients with high and low serum zinc levels and analyzed the risk factors related to low zinc level.	Lower serum zinc level in T2D patients was related to higher prevalence of diabetic microvascular complications, and represented as an independent risk factor for DN. Patients with lower zinc level were more likely to have a longer duration of diabetes, poorer glucose control, and worse β-cell function.	Low

#### 3.1.3. Fiber and hydratation, and DR

The keywords used in this research were: “fiber” OR “hydration status” OR “water intake” AND “DR” OR “eye diseases” OR “diabetes.” Six articles were sourced: one narrative review, one systematic review, two cross-sectional studies, one randomized controlled trial, and one *post-hoc* analysis of a randomized trial.

Table 3 includes studies that assessed the connection between fiber and hydration, and DR alongside the strength of evidence.

**Table 3:** Fiber, hydratation, and DR.

**References**	**Study design**	**Study period**	**Endpoint**	**Subjects (age, sex, number…)**	**Method**	**Results**	**Strength of evidence**
Diaz- Lopez et al. (30)	*Post-hoc* analysis of a randomized trial	6 years (from 2003 to 2009)	To determine the effect of the three dietary interventions on the incidence of diabetes complications.	Type 2 diabetes participating in the “PREvencion con DIeta MEDiterranea (PREDIMED)” randomized clinical trial, who were free of microvascular complications at enrolment (n = 3,614, aged 55–80 years)	Patients were randomly assigned to one of three dietary interventions: MedDiet supplemented with extravirgin olive oil (MedDiet+EVOO), MedDiet supplemented with mixed nuts (MedDiet+Nuts), or a low-fat control diet. Two independent outcomes were considered: new onset of diabetic retinopathy and nephropathy. Hazard ratios (HRs) were calculated using multivariable-adjusted Cox regression.	Compared with the control diet, multivariable-adjusted HRs for diabetic retinopathy were 0.56 (95% CI 0.32–0.97) for the MedDiet + EVOO and 0.63 (0.35–1.11) for the MedDiet+Nuts. No between-group differences were found for nephropathy.	Moderate
Salas-Salvadó et al. (31)	Randomized controlled trial	4 years	To test the effects of two Mediterranean diet (MedDiet) interventions vs. a low-fat diet on incidence of diabetes.	418 non-diabetic subjects aged 55–80 years recruited in one center of the Prevención con Dieta Mediterránea [PREDIMED] study, a large nutrition intervention trial for primary cardiovascular prevention in individuals at high cardiovascular risk.	Participants were randomly assigned to education on a low-fat diet (control group) or to one of two MedDiets, supplemented with either free virgin olive oil (1 liter/week) or nuts (30 g/day). Diets were *ad libitum*, and no advice on physical activity was given. The main outcome was diabetes incidence diagnosed by the 2009 American Diabetes Association criteria.	The Mediterranean diet, in fact, rich in food sources of fiber, such as fruit, vegetables and unrefined carbohydrates, has been associated with a reduced incidence of DR.	High
Zhang et al. (60)	Cross-sectional study	3 years	To explore the association between hydration status and DR.	5,220 US adults 40 years of age and older (2005–2008 NHANES study)	Serum osmolality was used to assess hydration status for all participants and calculated osmolality was evaluated for only older people.	Adults with lower hydration status had higher risk of DR, moderate/severe non-proliferative retinopathy, and proliferative diabetic retinopathy. Dehydration in older adults, classified by calculated osmolality, is associated with a higher rate of DR.	Low

#### 3.1.4. Gut microbiota and DR

These keywords were used as the basis for the research: “Gut microbiota” OR “dysbiosis;” AND “diabetic retinopathy” OR “eye diseases” OR “diabetes.” Four articles were sourced: one narrative review, two studies on animal models, and one comment on a study based on animal models.

#### 3.1.5. PA and DR

This study was done based on the following keywords: “physical activity” OR “sedentary behavior” OR “lifestyle” OR “resistance training” OR “aerobic exercise” AND “diabetic retinopathy” OR “eye diseases” OR “diabetes.” Twenty-four articles were sourced: four narrative reviews, three systematic reviews and meta-analysis, one review of *in vitro* studies, one mini review, nine clinical trials (one cross-sectional study, one retrospective study, one cohort study, three prospective trials, and three observational studies), five studies on animal models, and one Clinician's Guide.

The studies that assessed the connection between PA and DR are listed in Table 4 along with their strength of evidence.

**Table 4:** Physical activity (PA) and DR.

**References**	**Study design**	**Study period**	**Endpoint**	**Subjects (age, sex, number…)**	**Method**	**Results**	**Strength of evidence**
Praidou et al. (61)	Cross-sectional, non-interventional study	1 years	to investigate potential correlation between physical activity and diabetic retinopathy	320 patients were included in the study: 240 patients with diabetes type 2 (80 patients with mild to moderate NPDR, 80 patients with severe to very severe NPDR and 80 ones with PDR) were compared with 80 non-diabetic patients (control group).	Physical activity of patients was assessed by the international physical activity questionnaire. HbA1c and BMI were also measured in diabetic patients. Group comparisons were attempted for levels of physical activity and sedentary behavior.	Increased physical activity is associated with less severe levels of diabetic retinopathy, independent of the effects of HbA1c and BMI.	Low
Yan et al. (62)	Cohort study	10 years	To examine the association of physical activities (PA) with diabetic retinopathy (DR) progression based on a 10-year follow-up of a large cohort of working-aged diabetic populations in Australia.	Nine thousand and eighteen working-aged diabetic patients were enrolled from the baseline of the 45 and Up Study from New South Wales, Australia.	Self-reported PA collected by questionnaire at baseline in 2006 was graded into low (<5 sessions/week), medium (≥5 to 14), and high (≥14) levels. Retinal photocoagulation (RPC) treatment during the follow-up period was used as a surrogate for DR progression and was tracked through the Medicare Benefits Schedule, which was available from 2004 to 2016. Cox regression was used to estimate the association between PA and RPC incidence.	Higher PA level was independently associated with a lower risk of DR progression among working-aged diabetic populations in this large cohort study.	Low
Kuwata et al. (63)	Prospective observational study	2 years	To assess the association between baseline levels of physical activity (PA) and the incidence of newly developed diabetic retinopathy (DR) in patients with type 2 diabetes.	1,814 patients (mean age 65.5 years) with type 2 diabetes without DR were obtained from a Japanese diabetes registry at Tenri Hospital, Nara, Japan.	The participants were divided into five categories based on their PA levels. A Cox proportional hazards model with time-varying exposure information was used and adjusted for potential confounders to assess the independent correlations.	higher PA was independently associated with a lower incidence of DR in patients with type 2 diabetes.	Low
Al-Othman et al. (64)	Observational study	/	To determine whether the prevalence of vitamin D deficiency is related to degree of physical activity and sun exposure among apparently healthy Saudi children and adolescents.	331 Saudi children aged 6–17 years (153 boys and 178 girls)	Levels of physical activity and sun exposure were determined using a standard questionnaire. Anthropometry, serum calcium and 25-(OH) vitamin D were analyzed.	Vitamin D deficiency is common among Saudi children and adolescents, and is influenced by both sun exposure and physical activity. Promotion of an active outdoor lifestyle among Saudi children in both homes and schools may counteract the vitamin D deficiency epidemic in this vulnerable population.	Low
Scott et al. (65)	Prospective study	mean follow-up of 2.6 ± 0.4 years	To verify prospective associations between 25OHD, muscle parameters, and PA in community-dwelling older adults.	Six hundred and eighty-six community-dwelling older adults (49% women; mean ± SD 62 ± 7 years old).	Appendicular lean mass percentage (%ALM) and body fat assessed by Dual-energy X-ray Absorptiometry, leg strength by dynamometer, leg muscle quality (LMQ), PA assessed by pedometer, self-reported sun exposure by questionnaire, and serum 25OHD measured by radioimmunoassay.	25OHD may be important for the maintenance of muscle function, and higher skeletal muscle mass and function as well as general PA levels may also be beneficial for 25OHD status, in community-dwelling older adults.	Low
Klenk et al. (66)	Observational study	1 week	To analyse the seasonal relationship of objectively measured physical activity with vitamin D status in older persons from Southern Germany (latitude: 48.4°N).	1,193 community-dwelling individuals aged ≥65 years (58% men)	Physical activity was assessed in participants over 1 week using a thigh-worn accelerometer. Furthermore, the 25-hydroxyvitamin D (25(OH)D) level was measured. Least-square means of 25(OH)D serum levels were calculated for quartiles of average daily walking duration stratified by season and adjusted for gender, age and body mass index.	Although a positive dose-response relationship was seen between walking duration and the 25(OH)D serum level for most seasons, vitamin D insufficiency was still very prevalent even in high-active persons during all seasons.	Low
Black et al. (67)	Prospective study	6 years	To investigate vitamin D status and predictors of serum 25-hydroxyvitamin D (25(OH)D) concentrations in adolescents.	1,045 Australian adolescents	Serum 25(OH)D concentrations were measured in the same participants at 14 and 17 years (*n* 1,045 at both time points). The authors examined the predictors of serum 25(OH)D concentrations, including sex, race, month of blood collection, physical activity, BMI, family income, and Ca and vitamin D intakes (*n* 919 at 14 years; n 570 at 17 years), using a general linear mixed model.	The authors identified physical activity as a significant predictor of serum 25(OH)D concentrations in 258 adolescents	Low
Herrmann et al. (68)	Observational study	5 years	To investigate the relationship between blood 25-hydroxyvitamin D (25OH-D) concentration and vascular disease risk in type 2 diabetes.	9,795 participants aged 50–75 years with type 2 diabetes from the Fenofibrate Intervention and Event Lowering in Diabetes (FIELD) trial	The relationships between blood 25OH-D concentration at baseline and the incidence of macrovascular (including myocardial infarction and stroke) and microvascular (retinopathy, nephropathy, neuropathy, and amputation) disease were analyzed with Cox proportional hazards models and logistic regression.	Low blood 25OH-D concentrations are associated with an increased risk of macrovascular and microvascular disease events in type 2 diabetes.	Low
Schneider et al. (69)	Retrospective study	10 years	To examine the effects of a program of diet and exercise on various metabolic and hemodynamic parameters, and to assess the ability of patients to perform physical training safely.	255 previously sedentary diabetic patients and 58 control subjects	A group of individuals with diabetes attempted to use regular exercise as part of their therapeutic management. The patients were followed for varying times, up to 1 yr after entry into the physical training component of a diabetes lifestyle modification program. Metabolic and hemodynamic parameters were collected.	Study suggests a program of regular aerobic training can be safely and effectively used in an outpatient population with diabetes mellitus for up to 3 mo.	Low

Table 5 shows the reviews about DR and DPs.

**Table 5:** Dietary and food patterns and DR.

**References**	**Study design**	**Purpose**	**Main findings**
Dow et al. (70)	Systematic review	To identify, summarize and interpret the literature on the association between the diet and dietary intakes of specific foods, nutrients, and food groups, and the risk of diabetic retinopathy.	Adherence to the Mediterranean diet and high fruit, vegetable and fish intake may protect against the development of diabetic retinopathy, although the evidence is limited. Studies concerning other aspects of the diet are not in agreement. The role of the diet in the development of diabetic retinopathy is an area that warrants more attention.
Wong et al. (71)	Systematic review	To study the associations between dietary intake and DR, with the primary goal of providing a comprehensive assessment of the existing knowledge on the topic.	No significant associations of carbohydrate, vitamin D, and sodium intake with DR were found. Associations of antioxidants, fatty acids, proteins and alcohol with DR remain equivocal. Dietary fiber, oily fish, a Mediterranean diet and a reduced caloric intake are associated with lower risk of DR.
Francisco et al. (3)	Narrative review	To discuss the impact of dietary patterns on the incidence and progression of age-related eye diseases, namely age-related macular degeneration (AMD), cataracts, diabetic retinopathy, and glaucoma.	The authors found strong evidence about dietary patterns in regard to AMD and some in cataract, but there is surprisingly little conclusive evidence linking specific dietary patters with DR and glaucoma. Across studies looking at AMD progression, there are consensus findings that adherence to a prudent dietary pattern, the Mediterranean diet, and the healthy eating index all protect against AMD and that the western dietary pattern can accelerate AMD progression.
Ros et al. (72)	Narrative review	To focused on the latest findings concerning health effects of walnuts and ALA and relevant micronutrients.	Walnuts have a high content of fiber, polyphenols, phytosterols, gamma-tocopherol, and mainly linolenic acid (ALA), as well as various minerals, which confer antioxidant, anti-inflammatory, cardio- and neuro-protective, antithrombotic, antiarrhythmic, hypocholesterolemic properties and regulation of the intestinal microbiota
Poulose et al. (73)	Narrative review	To review evidences for the beneficial effects of consuming a walnut-rich diet.	Polyphenolic compounds found in walnuts not only reduce the oxidant and inflammatory load on brain cells but also improve interneuronal signaling, increase neurogenesis, and enhance sequestration of insoluble toxic protein aggregates.
Valero-Vello et al. (74)	Systematic review	To identify he role of diet and nutrition in the eyes and vision, and the potential antioxidant, anti-inflammatory and neuroprotective effects of natural food (broccoli, saffron, tigernuts and walnuts), the MD and nutraceutic for patients at risk of vision loss.	Nut-enriched diet bring benefits in ocular diseases, such as glaucoma, DR and degenerative maculopathy, chronic pathologies of a degenerative nature for the ocular structures, which have common pathophysiological mechanisms, related precisely to oxidative stress and inflammation
Meng et al. (75)	Systematic review	To summarize and discusses the effects of tea against diabetes mellitus and its complications based on the findings from epidemiological, experimental, and clinical studies, with the special attention paid to the mechanisms of action.	Epidemiological studies found that drinking tea could reduce the risk of diabetes mellitus and diabetic complications. In addition, experimental studies have shown that tea could protect against diabetes mellitus and diabetic complications by improving insulin resistance, activating the insulin signaling pathway, playing an insulin-like role, improving oxidative stress, and alleviating inflammatory response. Further, tea has synergistic effects with certain antidiabetic drugs. Tea has been observed to act as a potent neuroprotector in the retina.
Natella et al. (76)	Narrative review	To examine the possibility that the pattern of coffee consumption could influence risk of type 2 diabetes, and to evaluate the possible relationship between coffee consumption and other risk factors associated with diabetes.	The studies conducted thus far provide a clear indication that healthy, habitual coffee drinkers are more protected from the risk of contracting diabetes than individuals who do not drink coffee. Long-term consumption of coffee is able to reduce oxidative stress. This could be due to the caffeine itself, which is considered an antioxidant, but also to other coffee components,
Akash et al. (77)	Narrative review	To explore and summarize the scientific literature on the potential effects of coffee consumption on T2DM.	Coffee may directly affect different mechanistic factors that are involved in the pathogenesis of T2DM. Several components of coffee may ameliorate the symptoms of T2DM by affecting glucose regulation. These may include the effects of CGA on glucose-6-phosphatase, the antioxidant activity of polyphenols on α-glucosidase, and the effects of caffeine on insulin secretion.
Carlström et al. (78)	Meta-analyses of observational studies	To cover current knowledge regarding the effects of coffee consumption on development of T2D or modulation of adverse complications. Moreover, bioactive components in coffee, polymorphisms, and potential underlying mechanisms in relation to T2D and adverse complications are discussed.	Available evidence indicates that coffee consumption is inversely associated with risk of T2D. Possible mechanisms behind this association include thermogenic, antioxidative, and anti-inflammatory effects; modulation of adenosine receptor signaling; and microbiome content and diversity.

### 3.2. AMD

This research was conducted based on the keywords: “AMD” OR “AMD” AND “diet” OR “nutrients” OR “nutrition” OR “food” OR “supplements” OR “supplementation.” Nineteen articles were sourced: one randomized controlled trial, one cross-sectional trial, seven cohort studies, two multi-center studies, four population-based prospective studies, three case–control studies, and one clinical trial.

Table 6 includes the research that assessed the connection between AMD nutrition, including supplement use, and their level of evidence.

**Table 6:** Nutrition and AMD.

**References**	**Study design**	**Study period**	**End point**	**Subjects (age, sex, number…)**	**Method**	**Results**	**Strength of evidence**
Gopinath et al. (55)	Cohort study	2 years	Association between intakes and serum concentrations of folate and vit B12 or serum tHcy and 10-y AMD incidence	1,760 subjects aged ≥ 55 years	Serum folate, vit B12, and tHcy determined from blood samples drawn	Elevated serum tHcy and folate and vit B12 deficiencies predicted increased risk of incident AMD, suggesting a potential role for vit B12 and folate in reducing AMD risk	Low
Seddon et al. (79)	Cross-sectional study	/	Modifiable risk and protective factors for AMD among elderly twins	681 twins (222 with intermediate or advanced stage AMD and 459 without AMD or with initial signs of the disease)	Eye examination, fundus photography, food frequency and risk factor questionnaires. Risk for AMD according to cigarette smoking and dietary fat intake estimated using logistic regression analyses.	Cigarette smoking increases risk while fish consumption and omega-3 fatty acid intake reduce risk of AMD	Low
Chua et al. (80)	Cohort study	5 years	Associations between dietary fat and incident age-related maculopathy (ARM)	2,335 subjects aged ≥ 49 years	Semiquantitative food frequency questionnaire	Participants in the top quintile of omega-3 fatty acid intake had a lower risk of early AMD onset than the lowest quintile, with a 40% reduction in incidence when consuming fish at least once a week. No association between incident ARM and butter, margarine, or nut consumption.	Low
Tan et al. (81)	Cohort study	5 and/or 10 years	Relationship between baseline dietary fatty acids and 10-year incident AMD	3,654 participants examined at baseline and 2,454 examined 5 and/or 10 years later	Evaluation of AMD from retinal photographs; semiquantitative food frequency questionnaire	Evidence of protection against early AMD from regularly eating fish, greater consumption of omega-3 polyunsaturated fatty acids, and low intakes of foods rich in linoleic acid. Association between consumption of 1–2 portions of nuts per week (compared to less than one portion per week) and a reduction in the risk of early AMD.	Low
SanGiovanni et al. (82)	Case-control study (AREDS study)	/	Association of lipid and vit C intake with baseline severity of AMD	4,519 subjects (60–80 years of age at enrolment)	Stereoscopic color fundus photographs; estimates of habitual nutrient intake through self-administered semi-quantitative food frequency questionnaires	Top quintile of total long-chain omega-3 intake and DHA associated with a lower risk of neovascular AMD (NV AMD) than the bottom quintile. Higher consumption of fish inversely related to NV AMD; arachidonic acid taken with food directly associated with the incidence of this pathology. Reduced probability of developing neovascular AMD in subjects with the highest vitamin C intake (not confirmed following addition of covariates).	Low
Robman et al. (83)	Cohort study	7 years	Effects of dietary intake of lutein, zeaxanthin and fats on the progression of AMD	252 subjects (134 F−118 M) diagnosed with early AMD	Food frequency questionnaires to estimate the intakes of lutein, zeaxanthin and fatty acids	Association of increased intakes of dietary lutein, zeaxanthin and omega-3 fatty acids with progression of AMD, suggesting that too much of a good thing might be harmful.	Low/Moderate
Delcourt et al. (84)	Population-based study	1 year	Correlation between fats and increased risk of age-related maculopathy	832 subjects ≥70 years	Dietitian-administered food-frequency questionnaire	Association of high total, saturated and monounsaturated fat intake with increased risk for ARM. Total polyunsaturated fatty acid not significantly associated with ARM. Total and white fish intake not significantly associated with ARM; association of fatty fish intake (more than once a month vs. less than once a month) with a 60% reduction in risk for ARM	Low
Seddon et al. (85)	Multicenter case-control study	1 year	Relationship between intake of total and specific types of fat and risk for AMD	349 cases (age 55–80 years), affected by advanced neovascular AMD, and 504 controls without AMD but with other ocular diseases	Standardized interview, physical examination, ophthalmic examination, laboratory analysis of blood specimens, semi- quantitative food-frequency questionnaire (a modification of an extensively validated questionnaire, containing a list of food items selected as the major sources of a variety of nutrients and adapted for use among elderly subjects with eye disease)	Higher intake of specific types of fat (including vegetable, monounsaturated, and polyunsaturated fats and linoleic acid) rather than total fat intake associated with greater risk for advanced AMD. Inverse association between diets high in omega-3 fatty acids and fish and risk for AMD if low intake of linoleic acid.	Low
Chiu et al. (86)	Clinical trial	8 years	Effects of AREDS supplement, intake of docosahexaenoic acid (DHA) and eicosapentaenoic acid (EPA), and dietary glycaemic index (dGI) on AMD	2,924 subjects, mean age 69.3 years, 1,698 F−1,226 M	Validated 90-item modified Block FFQ; stereoscopic fundus photographs of the macula.	Association of higher intakes of DHA, EPA and lower dGI with a lower risk for progression to advanced AMD.	Moderate
Seddon et al. (87)	US multicentre study EDCCS (Eye Disease Case-Control Study)	/	Association of blood levels of carotenoids and dietary carotenoids and vitamins C, A and E intake with risk of AMD.	421 patients with neovascular AMD and 615 controls	Blood analysis; surveys.	Markedly reduced risk (equal to one-half and one-third, respectively) in subjects with intermediate and high blood levels of carotenoids compared to participants with low levels; subjects in the top quintile of dietary carotenoid intake with a risk of AMD 43% lower than subjects in the bottom quintile (lutein and zeaxanthin associated with the strongest reduction of AMD risk). No statistically significant association between consumption of vitamin C and risk of AMD.	Low
Snellen et al. (88)	Case-control study	/	Association of low antioxidant intake with the occurrence of AMD	72 cases and 66 control patients	Interview on antioxidant intake (i.e., in fruit and vegetables), cigarette smoking, sunlight exposure and familial predisposition. Antioxidant intake calculated according to the method described in the Framingham Eye Study.	Prevalence rate of AMD in patients with low antioxidant intake and low lutein intake (dichotomized at the median value) about twice as high as that in patients with high intake, with a clear dose–response relationship.	Low
Goldberg et al. (27)	NHANES study	2 years	Determination of factors associated with the prevalence of AMD.	Subjects aged ≥45 years at the time of the ophthalmology examination	Ophthalmology examination; FFQ.	Negative association of the frequency of consumption of fruits and vegetables rich in vitamins A and C with the prevalence of AMD after stratified adjustment for age.	Low
Simonelli et al. (89)	Case-control study	/	Determination of the weight of oxidative status as risk factor in the early stage of AMD.	Forty-eight ARM patients (19 early and 29 late form) and 46 normal subjects	Determination of serum/plasma antioxidants (vitamins C, E, A, total and individual carotenoids, zinc, total plasma antioxidant capacity— TRAP) and oxidative parameters (reactive oxygen metabolites—ROM, oxidized-low-density lipoprotein antibodies—anti-Ox- LDL).	Association of a deficit of antioxidants (vitamins C, E and carotenoids) with AMD in Italian patients, particularly the advanced form (in AMD patients macular susceptibility to oxidative damage not related with age).	Low
Kassoff et al. (90)	11- center, randomized, placebo-controlled, double-masked clinical trial (AREDS)	Average follow-up of 6.3 years	Photographic assessment of progression to or treatment for advanced AMD and at least moderate visual acuity loss from baseline.	3,640 participants, aged 55–80 years (3416 M−224 F)	Participants randomly assigned to receive daily oral tablets containing: (1) antioxidants (vitamin C, 500 mg; vitamin E, 400 IU; and beta carotene, 15 mg); (2) zinc, 80 mg, as zinc oxide, and copper, 2 mg, as cupric oxide; (3) antioxidants plus zinc; or (4) placebo.	Statistically significant reduction fof the development of advanced AMD with antioxidants plus zinc. Association of both zinc and antioxidants plus zinc with significantly reduced odds of developing advanced AMD. No statistically significant serious adverse effect associated with any of the formulations.	High
Klein et al. (91)	Population-based prospective study (Beaver Dam Eye Study)	15 years	Incidence of age-related cataracts, AMD, and high IOP for one set of analyses and incidence of supplement use for the second set of analyses.	Participants in the Beaver Dam Eye Study contributing data in 1988 to 1990 (*n* = 4,926), 1993 to 1995 (*n* = 3,722), 1998 to 2,000 (*n* = 2,962), and 2003 to 2005 (*n* = 2,375).	Data about use of all medications and supplements collected from participants at each of 4 examinations; intraocular pressure (IOP) measurement and fundus and lens photography at each visit; visual field data available only from baseline; photographs of the lenses, retina, and discs graded using standard protocols by trained graders.	Little evidence of any significant associations between supplement use and incident ocular outcomes except for a small protective effect for cortical cataracts by vitamins A and D, zinc, and multivitamins and increased odds of late AMD. Association of late AMD with incident use of vitamins A, C, and E and zinc.	Low
Merle et al. (92)	Prospective cohort study	13 years of follow-up	Association of adherence to the Mediterranean diet and genetic susceptibility with progression to advanced AMD	2,525 subjects (1,124 M−1401 F)	Demographic questionnaires; FFQ; the alternate Mediterranean diet (aMeDi) score; examination of ten genetic loci in 7 genes.	Association of a higher adherence to a Mediterranean diet with reduced risk of progression to advanced AMD, modifiable by genetic susceptibility.	Low
de Koning-Backus et al. (93)	Prospective population-based cohort study	9.1 ± 5.8 years of follow-up	Association of the intake of vegetables, fruit, and fish with incident AMD.	4202 participants from the Rotterdam Study (≥55 years of age)	Fundus photographs; validated 170-item FFQ (food intakes categorized into food patterns based on guidelines from Health Council).	Association of a diet of 200 grams per day of vegetables, fruit two times per day, and fish two times per week with a significantly reduced risk of AMD.	Low
Chiu et al. (94)	/	/	Relationship of predominant dietary patterns with AMD	A total of 8,103 eyes from 4,088 participants	Using principle component analysis, characterization of major and minor dietary patterns; logistic regression.	Association of the two major patterns (Oriental and Western) with both early and advanced AMD; no association of minor patterns with early AMD; only four of these significantly associated with advanced AMD, including Steak pattern, Breakfast pattern, Caribbean pattern and Peanut pattern.	Low
Amirul Islam et al. (95)	Cohort study	9–17 years of follow-up	Odds ratios for early stages and advanced AMD in association with dietary patterns	21,132 participants, aged 40 to 70 years	Retinal photographs; FFQ; principal component analysis used to identify dietary patterns; logistic regression.	Association of a dietary pattern high in fruits, vegetables, chicken, and nuts and a pattern low in red meat with a lower prevalence of advanced AMD; no association of a particular food pattern with the prevalence of the earliest stages of AMD.	Low

### 3.3. Cataracts

These keywords served as the basis for the research: “cataract” OR “lens opacities” AND “nutrition” OR “supplementation” OR “supplementation” OR “physical activity” OR “hydration.” Eleven articles were sourced: Four observational studies, four case–control studies, one narrative review, one systematic review, and one meta-analysis.

Tables 7, 8 include the research that evaluated the relationship between PA and bone along with the strength of the evidence.

**Table 7:** Nutrition and cataract (observational studies and case-control studies).

**References**	**Study design**	**Endpoint**	**Study period**	**Subjects (age, sex, number…)**	**Method**	**Results**	**Strength of evidence**
Christen et al. (96)	Observational study	to examine whether higher fruit and vegetable intake reduces the risk of cataract and cataract extraction in a large, prospective cohort of women.	8 years of medium follow up	39,876 apparently healthy female health professionals aged ≥45 y	Fruit and vegetable intake was assessed at baseline with the use of a validated, semiquantitative food-frequency questionnaire. A total of 35 724 of these women were free of a diagnosis of cataract at baseline and were followed for incident cataract and cataract extraction.	During an average of 10 y of follow-up, 2,067 cataracts and 1,315 cataract extractions were confirmed. Compared with women in the lowest quintile of fruit and vegetable intake, women with higher intakes had modest 10–15% reduced risks of cataract (*P* for trend < 0.05).	Moderate
Jacques et al. (97)	Observational study	To assess the relation between usual nutrient intake and subsequently diagnosed age-related nuclear lens opacities	15 years	Four 478 non-diabetic women aged 53–73 years without previously diagnosed cataracts sampled from the Nurses' Health Study cohort	Usual nutrient intake was calculated as the average intake from 5 food frequency questionnaires that were collected during a 13- to 15-year period before the evaluation of lens opacities. The duration of vitamin supplement use was determined from 7 questionnaires collected during this same period	vitamin C intake remained significantly associated (*P* = 0.003 for trend) with the prevalence of nuclear opacities. The prevalence of nuclear opacities was significantly lower (*P* < 0.001) in the highest vitamin C intake quintile category relative to the lowest quintile category (odds ratio, 0.31; 95% confidence interval, 0.16–0.58). There were also statistically significant trends of decreasing prevalence of nuclear opacities with increasing duration of use of vitamin C (*P* = 0.004 for trend).	MODERATE
Delcourt et al. (84)	Observational study	To assess the associations of plasma lutein and zeaxanthin and other carotenoids with the risk of age-related maculopathy (ARM) and cataract in the population-based Pathologies Oculaires Liées à l'Age (POLA) Study	–	2,584 participants, age over 60 years	Cataract classification was based on a direct standardized lens examination at the slit lamp, according to Lens Opacities Classification System III. Plasma carotenoids were measured by high-performance liquid chromatography (HPLC), in 899 subjects of the cohort.	After multivariate adjustment, the highest quintile of plasma zeaxanthin was significantly associated with reduced risk of nuclear cataract (OR = 0.23; 95% CI: 0.08–0.68; *P* for trend = 0.003) and any cataract (OR = 0.53; 95% CI: 0.31–0.89; *P* for trend = 0.01). Among other carotenoids, only β-carotene showed a significant negative association with nuclear cataract.	Moderate
Moeller et al. (98)	Observational study	to evaluate associations between nuclear cataract (determined from slitlamp photographs between May 2001 and January 2004) and lutein and zeaxanthin in the diet and serum	–	total of 1,802 women aged 50 to 79 years with intakes of lutein and zeaxanthin above the 78th (high) and below the 28th (low) percentiles in the Women's Health Initiative Observational Study (1994–1998) were recruited 4 to 7 years later (2001–2004) into the Carotenoids in Age-Related Eye Disease Study.	Serum samples were obtained from participants at WHI baseline examinations (1994–1998) after a ≥ 10-h fast and were stored at −80 degrees centigrade 0.29 Serum levels of lutein, zeaxanthin, and tocopherols were determined at Tufts University (2004–2005) by a reverse phase HPLC analysis. Measurements were made using a standardized protocol by the psychophysical method of heterochromatic flicker photometry (HFP) during CAREDS study visits conducted from 2001 to 2004. This protocol, described in detail previously, 20, 33 had high test-retest reliability (*r* = 0.9) and participant response. *T*-tests, ANCOVA, and Chi-Square tests were performed to assess the statistical significance of potential covariates	Women in the group with high dietary levels of lutein and zeaxanthin had a 23% lower prevalence of nuclear cataract (age-adjusted odds ratio, 0.77; 95% confidence interval, 0.62–0.96) compared with those with low levels.). Women in the highest quintile category of diet or serum levels of lutein and zeaxanthin as compared with those in the lowest quintile category were 32% less likely to have nuclear cataract (multivariable-adjusted odds ratio, 0.68; 95% confidence interval, 0.48–0.97; *P* for trend = 0.04; and multivariable-adjusted odds ratio, 0.68; 95% confidence interval, 0.47–0.98; *P* for trend = 0.01, respectively).	Moderate
Pastor-Valero et al. (99)	Case-control study	To Investigate the association of antioxidant vitamins and minerals and risk of cataract in a Mediterranean population	-	Cases with cataract (343) and 334 age/sex frequency-matched controls aged 55 to 74 y were selected from an ophthalmic outreach clinic	Participants were interviewed about their diet using a Food Frequency Questionnaire, and other information on potential confounders, such as smoking, alcohol, and education. Blood samples were analyzed by a colorimetric method for vitamin C and by reversed-phase HLPC for other blood antioxidants	Blood levels of vitamin C above 49 micromol/L were associated with a 64% reduced odds for cataract (*P* < 0.0001). Dietary intake of vitamins C, E and selenium were marginally associated with decreased odds (*P* = 0.09, *P* = 0.09, *P* = 0.07, respectively), whereas moderately high levels of blood lycopene (>0.30 micromol/L) were associated with a 46% increased odds of cataract (*P* = 0.04). results strengthen the evidence for a protective role for vitamin C on the aging lens as this effect was seen in a population characterized by high vitamin C intakes	Moderate
Ghanavati et al. (100)	case-control study	To evaluate and compare healthy eating index among the patients with cataract and healthy individual	–	97 patients with cataract and 198 healthy people (as a control group)	Individuals were selected by the convenience sampling method and the food frequency questionnaire (FFQ) was completed for them. At first, HEI was calculated and then the HEI scores were compared in cataract patients and healthy individuals.	The analysis of FFQ showed that the scores of vegetables (7.81 vs. 10), nutritional variation (5.5 vs. 7) and sodium (2 vs. 6) groups (*P* < 0.001) were significantly lower among the patients with cataract than the healthy individuals. Also this significant difference was observed in the scores of total HEI and fruits (respectively 73.26 vs.79.30 and 7.1 vs. 9.8) (*P* < 0.01). On the other hand, the scores of saturated fatty acids (10 vs. 9; *P* = 0.02), total fat (8 vs. 7; *P* = 0.004) and cereals (10 vs. 10; *P* < 0.001) were higher among the patients with cataract than the healthy individuals. In addition, after adjusting the confounding factors the results showed that the HEI high score was associated with reducing the risk of coming down with cataract (OR = 0.18, CI: 95%, *P* < 0.001, 0.08–0.41).	Moderate
Sedaghat et al. (101)	case-control study	To assess the relation between nutrient patterns and cataract risk	–	97 cataract patients and 198 matched controls.	Dietary consumption was collected through a valid food frequency questionnaire (FFQ). Nutrient patterns were detected by applying factor analysis. Unconditional logistic regression models were used to estimate odds ratio (ORs) and 95%Cis. They identified 5 main nutrient patterns: -Pattern 1: included niacin, thiamin, carbohydrates, protein, zinc, vitamin B6 and sodium (sodium pattern) -Pattern 2: characterized by oleic acid, monounsaturated fats, polyunsaturated fats, linoleic acid, trans fatty acid, linolenic acid, vitamin E and saturated fats (fatty acid pattern). -Pattern 3: factor represented high intake of vitamin B12, vitamin D, cholesterol and calcium (mixed pattern) -Pattern 4: high in intake of beta and alpha carotene, vitamin A and vitamin C (antioxidant pattern). - Pattern 5: pattern loaded heavily on docosahexaenoic acid (DHA) and eicosapentaenoic acid (EPA) (omega-3 pattern)	In crude and multivariate analysis, the sodium pattern was associated with increased risk of cataract (OR = 1.97, 95%CI: 1.09–3.96). The fatty acid pattern elevated the risk of cataract (OR = 1.94, 95%CI: 1.1–3.86). Antioxidant pattern was associated with a significant 79% reduced risk (2nd category compared with the 1st). Omega-3 pattern was significantly negatively associated with risk of cataract (*P* = 0.04).	Moderate
Minassian et al. (102)	case-control study	To investigate the association between cataract and clinical manifestations concearning hydratation	24 months	1,364 patients, 881 males, 483 females. Divided in cataract and non-cataract group depending on ophthalmic diagnosis	All patients aged 30 to 69 attending the eye unit during the study period were systematically tested and examined to assess visual acuity and central lens opacities. Generalities and antropometry were assessed. All patient underwent an interview about past risk factor and past diseases concearning hydratation.	The results strongly confirm the findings from the first study and indicate thatan estimated 38% of blinding cataract may be attributable to repeated de-hydrational crises resulting from severe life threatening diarrhoeal disease and/or heatstroke. The risk of blinding cataract was strongly related to level of exposure to de-hydrational crises in a consistent and dose-dependent manner, thus indicating a causal association.	Moderate

**Table 8:** Nutrition and cataract (reviews).

**References**	**Study design**	**Purpose**	**Main findings**	**Strength of evidence**
Chong and Wong (103)	Narrative review	To examine literature evidences about cataract and nutrition	Dietary modifications that can retard cataract formation, if found, can have pro- found implications by reducing the personal, community, and financial burden caused by this common condition. However, based on the literature available currently, definitive recommendations on the use of a multivitamin supplement in preventing age-related cataract are premature	Low
Jiang et al. (104)	Meta-analysis	To summarize quantitatively the prospective association between physical activity and age-related cataract (ARC) risk	The findings from this Meta-analysis provide additional evidence that increased physical activity is inversely associated with age-related cataract risk dose-responsively	High
Sherwin et al. (105)	Systematic review	To investigate current evidence implicating changes in hydration and their association with ocular physiology and morphological characteristics and to asses relevant clinical correlations of changes in hydration and major common eye diseases	systemic hydration status broadly affects a variety of ocular pathophysiologic processes and disease states and the assessment of hydration status may be an important consideration in the management of patients with chronic eye diseases.	High

## 4. Discussion

### 4.1. DR

DR is a major microvascular complication of diabetic disease and is a major cause of vision loss in working-age populations globally (106–108). In a meta-analysis of 35 studies conducted worldwide between 1980 and 2008, an overall prevalence of DR of 34.6% (95% CI 34.5–34.8), proliferative DR (PDR) of 6.96% (6.87–7.04), and diabetic macular edema (DME) of 6.81% (6.74–6.89) was recorded; it has also been estimated that 10.2% (10.1–10.3) of diabetic patients are at risk of visual impairment from retinopathy (107). Complex microvascular, neurodegenerative, immunological, genetic/epigenetic, and inflammatory interactions contribute to the development of DR (109). Among the various factors involved, there are both modifiable and non-modifiable risk factors. Modifiable risk factors include hyperglycemia, arterial hypertension, dyslipidemia, obesity and inadequate nutritional status, hyperhomocysteinemia, chronic kidney disease, alcohol consumption, and smoking. Those that cannot be modified are represented by gender, age, myopia, duration of the diabetic disease, type of diabetes, and family history of DR (110, 111).

This broad range of pathogenic pathways explains how hyperglycemia is etiologically related to aging and other pathologies, including DR and AMD. Therefore, in this context, these pathologies can be considered metabolic diseases of the retina in all aspects (112).

Obesity is frequently linked to DM and cardiovascular disease as a risk factor. It can be defined by waist-to-hip ratio, waist circumference, and body mass index (BMI). Both higher waist-to-hip ratio and waist circumference are risk factors for DR (113–115). The OR of DR is 1.28 per 5 cm increase in waist circumference (OR = 1.28; 95% CI, 1.05–1.56; *P* = 0.014) (115). Also, malnutrition is a potential risk factor for the development of DR (116).

#### 4.1.1. Hyperhomocysteinemia

The enzyme methylenetetrahydrofolate reductase (MTHFR) is essential for adding the methyl group to folates. Polymorphisms in the MTHFR gene that reduce its activity, impairing the enzyme's ability to generate L-methylfolate, are common (117). These mutations are associated with hyperhomocysteinemia and other diseases, including DR (118, 119). At the cellular level, it has been demonstrated that a high level of homocysteine is harmful to the hemo-retinal barrier and has a pro-inflammatory effect on the epithelial cells of the retinal pigment, with the risk of increasing apoptosis phenomena (120). Elevated homocysteine levels increase the risk of hypertension, hypertensive retinopathy, diabetes, and DR (121) and are also associated with increased incidence and progression of DR (118, 119).

Supplementation with L-methylfolate [the bioactive form of folic acid (118)] can lead to the conversion of homocysteine into methionine, restoring its stocks, regardless of dietary deficiencies or genetic polymorphisms (122, 123). Optimal combinations of vitamins B1, B2, B6, L-methylfolate, methylcobalamin (B12), C, D, natural vitamin E complex, lutein, zeaxanthin, and alpha-lipoic acid are identified for protecting the retina and choroid. Nutritional interventions can support conventional therapies for DR to reduce the disease risk and severity of DR (122).

As far as alcohol is concerned, two important publications in literature have dealt with the relationship between DR and the consumption of alcoholic beverages (124, 125). Both concluded that there was no statistically significant association between alcohol consumption and DR risk. A first meta-analysis was conducted by Zhu in 2017 and included a total of 15 studies. Interestingly, in the statistical analysis analyzing different types of alcoholic beverages, wine or sherry intake was associated with a reduced risk of DR. In the publication, however, it was not possible to establish the dose responsible for this reduction, since there were no statistically significant differences between the various quantities taken (124, 125). The authors attributed this result to the potential protective effects of consuming low-to-moderate alcohol levels on the risk of diabetes mellitus (DM) and cardiovascular disease (126). However, the inflammatory response and oxidative stress could be influenced by alcohol, and are significantly associated with the risk of DR (127, 128). The stratified analyses of this meta-analysis were mixed due to the presence of various types of included studies; therefore, the results of these analyses are unreliable. A second meta-analysis in 2020 by Chen undertaken to correct the previous one and to implement the analysis with the new works that had been published in the meantime, confirmed the results of the previous one, not finding any significant association between alcohol intake and risk of DR of alcoholic beverages (125). However, even in this case, most of the studies considered reported inconsistent results. The Casteldaccia study showed that the duration of alcohol intake between 1 and 19 years was not associated with a risk of DR, but conversely, there was a reduction in the risk of DR with alcohol intake for a greater or equal number of years at 20 (129). According to Beulens' study, people with type 1 diabetes who drink moderate amounts of alcohol had a lower risk of microvascular problems (130). Fenwick showed that people with type 2 diabetes who occasionally consumed white wine had a lower risk of developing diabetic complications (131). This cross-sectional study was conducted in 2015 in patients with type 2DM, who were given a questionnaire that evaluated alcohol consumption and lifestyle. Patients included in the study then underwent retinography, and DR was staged as absent, present without, and at risk of vision loss. The relationship of DR intensity to alcohol consumption was adjusted for clinical-demographic, socioeconomic, and lifestyle factors. After adjusting for traditional risk factors and those for which they varied in univariate analysis, it was discovered that moderate drinkers (1–14 units/week) had a decreased risk of developing DR than non-drinkers. Therefore, the study concludes that in type 2 diabetics, the moderate consumption of alcoholic beverages is independently associated with a reduced risk of DR.

#### 4.1.2. Dietary and food patterns

Regarding DPs, there is evidence for the protective effect of the Mediterranean diet on the onset of DR. Diaz-López conducted a nutritional intervention study in type 2 DM patients who did not have microvascular complications at baseline. Three different dietary models were analyzed: the Mediterranean diet supplemented with extra virgin olive (EVO) oil, the Mediterranean diet supplemented with nuts, and a low-fat control diet. After a 6-year follow-up, it was observed that the Mediterranean diet supplemented with EVO oil had a protective effect on the development of DR (30). A 2018 review by Dow examined the association among individual foods, macro- or micronutrients, dietary supplements, DPs, and DR or DME. In particular, the following were taken into consideration: fruit, vegetables, fish, milk, carbohydrates, fibers, fats, proteins, salt, potassium, vitamins C, D, and E, carotenoids, food supplements, green tea, and alcohol. Studies suggest that adherence to the Mediterranean diet and a high intake of fruit, vegetables, and fish may protect against the development of DR, although evidence is limited (70). Another review, also published in 2018, systematically searched the literature for studies on diet and DR published between 1967 and 2017 using standardized criteria for diet and DR. The review concluded that higher dietary intake of fiber and fish and higher adherence to the Mediterranean diet were protective against DR. Conversely, high total caloric intake was associated with increased risk of DR. No significant association was found among carbohydrates, vitamin D, sodium, and DR; however, the association between DR and antioxidants, fatty acid (FA), proteins, and alcohol remained in doubt (71). In a more recent review, released in 2020, the effect of DPs on the occurrence and development of age-related eye illnesses such as DR, degenerative maculopathy, cataracts, and glaucoma was reviewed. Treatments for diabetes should slow the growth of DR. However, only a small number of research have confirmed if following particular DPs or eating a more or less healthy diet affects the prevalence of DR (3). For example, the randomized clinical trial PREDIMED (Prevention with Mediterranean Diet) (132) demonstrated how adherence to the Mediterranean diet could prevent diabetes. To see if DR might be avoided similarly, it seemed sense to do so. According to Dáz-post-hoc López's *post-hoc* analysis of the PREDIMED research, consuming 500 mg/day of omega-3 fatty acids (a readily feasible intake with strong adherence to the Mediterranean DP) considerably lowers the chance of getting DR (30).

Eating fish (an omega-3-rich dietary source) does, in fact, assist to delay the onset of DR. Eating oily fish at least two times weekly (rather than less frequently) has been related to a roughly 60% lower incidence of retinopathy (32). According to a 2017 cross-sectional study carried out in Palestine, a healthy eating pattern known as “Asian,” which is characterized by a high intake of whole grains, potatoes, legumes, vegetables, and fruit, can be linked to a lower prevalence of diabetes problems. This was contrasted with the “sweet-soft drinks-snacks pattern,” which was described as a harmful eating behavior characterized by high consumption of refined cereals, sugar, sweets, desserts, snacks, and soft drinks (33).

#### 4.1.3. Fruits and vegetables

In a Japanese cohort study that considered type 2 DM patients, a high fruit intake was linked to a decreased risk of DR. It was discovered that daily fruit eating of at least 173.0 g was related to a 50% lower risk of retinopathy incidence than daily fruit consumption of 53.2 g or less (34). Fruits and vegetables are generally good sources of flavonoids, fiber, minerals, and vitamins. They should be consumed in sufficient amounts, or at least 400 g per day, at each meal. The risk of cardiovascular events is decreased by 4% and the risk of stroke is decreased by 5% with each additional serving of fruit and vegetables (35).

#### 4.1.4. Nuts

Gamma-tocopherol, phytosterols, polyphenols, fiber, and linolenic acid (ALA) are all abundant in walnuts, as well as various minerals, which confer antioxidant, anti-inflammatory, cardio- and neuro-protective, antithrombotic, antiarrhythmic, hypocholesterolemic properties, and regulation of the intestinal microbiota (72). Nut consumption has been linked in human clinical trials to enhance cognitive function, with favorable effects on memory, learning, motor coordination, anxiety, and locomotor activity (36, 73). These researches also concluded that a diet high in nuts is beneficial for treating brain disorders and other chronic conditions linked to inflammation and oxidative stress (36, 37, 73). These health benefits also occur at the ocular level in various diseases, such as glaucoma, DR and degenerative maculopathy, and chronic pathologies of a degenerative nature for the ocular structures, which have common pathophysiological mechanisms related precisely to oxidative stress and inflammation (74).

#### 4.1.5. Saffron

In DR, saffron may reduce insulin resistance in patients with “prediabetes” (74). It has been shown *in vitro* that saffron can control the activation of microglia. Moreover, crocin (the carotenoid that gives saffron its distinctive color) supplementation reduces retinal thickness and enhances visual acuity in patients with diabetic macular edema, perhaps as a result of its anti-inflammatory effects (120).

This was observed in a double-blind, placebo-controlled, randomized phase 2 clinical trial. Sixty patients with diabetic maculopathy refractory to conventional therapy [including macular photocoagulation and intravitreal injection of an endothelial growth factor inhibitor (bevacizumab) with or without steroids (triamcinolone)] were considered. Patients were divided into three groups: patients in the crocin group were given 5 mg or 15 mg crocin tablets daily for 3 months, while patients in the placebo group received one placebo tablet daily during the study. Best corrected visual acuity (BCVA) and central macular thickness (CMT) were measured at baseline, and then monthly for a total of 3 months post-operatively. Blood chemistry tests were also evaluated at baseline and at the end of the study. BCVA and CMT were evaluated as primary outcomes, while glycated hemoglobin (HbA1c) and fasting blood glucose (FBS) were investigated as secondary outcomes in this study. The results showed that administering crocin tablets 15 mg daily could significantly reduce HbA1cg and CMT and improve BCVA compared to the placebo group. Although administering crocin tablets 5 mg daily can improve HbA1c, FBS, CMT, and BCVA, the difference was not significant compared with the placebo group. Thus, this study highlighted how crocin may act as a potent antioxidant and neuroprotective in short-term refractory DME; however, the clinical significance has yet to be demonstrated in a longer study with a larger sample size that includes treatment-naïve patients (120).

#### 4.1.6. Curcumin

Literature has shown that turmeric has an interesting activity on the retina; *in vitro*, treatment of high-glucose-induced human retinal endothelial cells (HRECs) with curcumin significantly reduced the intracellular production of reactive oxygen species (ROS), as well as the release of tumor necrosis factor-α (TNFα) (133).

Similar results were also obtained when curcumin was added to particular cell lines of the retinal pigment epithelium, called ARPE-19 (a spontaneous human retinal pigment epithelium cell line with normal karyotype that forms polarized epithelial monolayers on porous filter media) (134, 135).

Curcumin reduced the production of ROS and increased the expression of heme oxygenase-1 (HO-1), a type of redox-sensitive protein, whose activation protects cells from various types of stress. These findings imply that curcumin exhibits indirect antioxidant activity in addition to direct antioxidant activity by enhancing the activity of HO-1 and other antioxidant enzymes (134, 136).

Similarly, Maugeri argued that curcumin treatment can largely prevent the changes of DNA methyltransferase activity in high glucose-related stress ARPE-19 cells by downregulation of ROS production (137).

Given this background, although the studies are currently conducted exclusively *in vitro* and in the animal model, the results of the same encourage the routine intake of curcumin at least weekly.

#### 4.1.7. Tea and coffee

Tea has been found to act as a strong neuroprotector in the retina (75), inhibiting neovascularization and protecting pericytes preventing neovascularization (138). By lowering the production of ROS, boosting the expression of the glutamate transporter, reestablishing intercellular connections, and promoting glutamine/glutamate recycling, green tea can preserve retinal neurons in diabetes patients and control the retinal microenvironment (139). Furthermore, a low dose of green tea improves antioxidant defenses, reduces markers of inflammation, and prevents retinal basement membrane thickening (140). In a Chinese case–control study, including diabetic patients without DR, regular consumption of Chinese green tea every week for at least 1 year was associated with a reduced likelihood of DR in female subjects, but not in male subjects (38). In an animal model study, black tea was shown to lower blood sugar and slow the development of cataracts (141). Green and black tea (in 1.25% water) were administered to streptozotocin-induced diabetic rats for 3 months. Normal and diabetic control groups were also studied. As expected, the tested diabetic animals showed a significant increase in lens and plasma glucose. Red blood cell lens and sorbitol were significantly increased due to activation of the aldose reductase pathway. The thiobarbituric acid reactive substances of plasma, lens lipids, and protein glycation were also significantly elevated. Both teas significantly inhibited diabetic cataracts and caused significant reductions in the biochemical pathway implicated in the development of the disease. After corrections for glucose, teas have been found to delay the development of diabetic cataracts due to a hypoglycemic effect, which in turn inhibits biomarkers of the disease. Significant correlations were found among glucose level, cataract severity, and these indicators. Green tea, but not black tea, caused a significant drop in triglycerides in diabetic animals. The study concludes that tea may be a simple and cost-effective means of preventing or delaying diabetes in humans and resulting complications. Therefore, tea should also be studied as a therapeutic adjuvant in the treatment of diabetes. As for coffee, its long-term consumption can reduce oxidative stress (76). This could be due to the caffeine itself, which is considered an antioxidant, but also due to other coffee components, such as some trace elements (zinc, copper, and iron) and other substances, including chlorogenic acid (CGA), cafestol, trigonelline, and caffeic and ferulic acids (77). By modifying adenosine signaling, inhibiting glucose-6-phosphatase, inhibiting glucose-6-phosphate translocase 1, inhibiting intestinal glucose absorption, decreasing glucose production in the liver, increasing insulin secretion from pancreatic islets, and improving peripheral insulin sensitivity and glucose uptake, caffeine and CGA affect insulin and glucose homeostasis (by stimulation of the glucose transporter type 4 (GLUT4) and modulation of the activation of the intracellular signaling pathway that includes Akt, AMPK, and MAPK) (78). In healthy, obese, and 2DM adults, regular coffee consumption can reduce levels of pro-inflammatory biomarkers. The anti-inflammatory adiponectin, interleukin 4, and interleukin 10 can all be increased by it (76, 77). A Norwegian study found that high compared with low coffee consumption reduced the risk of type 2 DM by about 35% (39). This study looked at more than 360,000 subjects between 40 and 45 years of age, over 20 years, who were divided according to coffee consumption into four groups: <1 cup of coffee per day, 1 to 4 cups/day, 5 to 8, or more than 9 cups of coffee per day. The group that drank less than one cup of coffee per day was used as a reference. Compared to this, the other groups had relative risks of developing type 2 DM (0.87 for the 1–4 cups/day group, and 0.65 for both the 5–8 cups/day and > 9 cups/day groups). This regards the consumption of “boiled” coffee, while for other types of coffee, mainly filtered, the relative risks were as follows: 0.84 (1–4 cups/day), 0.67 (5–8 cups/day), and 0.62 (>9 cups/day). Similar results were obtained in a Finnish cohort study, in which coffee consumption was inversely correlated with type 2 DM (40).

#### 4.1.8. Nutrients

##### 4.1.8.1. Vitamins and antioxidants

It has been demonstrated that vitamins and antioxidants (such as vitamins C, E, and carotenoids) may play a role in the pathogenesis of DR as they lead to a reduction of retinal neovascularization, with the restoration of blood flow and have a protective role against free radicals (142). Furthermore, vitamins C and E appear to suppress vascular endothelial growth factor (VEGF) production in animal models and decrease advanced glycation end-products (AGEs) accumulation. Vitamin C can decrease protein kinase C activation (143), prevent glucose-induced pericyte apoptosis (144), and reduce oxidative stress in human retinal pigment epithelium (145). Given these premises, several studies have attempted to establish whether there was a relationship between DR and dietary antioxidant intake.

##### 4.1.8.2. Vitamin A and carotenoids

According to Brazionis, greater plasma levels of lutein and zeaxanthin were linked to a decreased risk of DR, just like they were for AMD (41). Taking lutein supplements at a level of 6 mg per day for 20 days per month (considered a “nutritional” intake, meaning typically ingested with a healthy and diverse diet) can stop the progression of DR within 5 years, according to a randomized trial on certain antioxidants (42). Patients with non-proliferative DR (NPDR) who take 10 mg of lutein daily report improved contrast sensitivity, glare, and visual acuity (43). In a 2-year study, diabetic individuals without DR who received 10 mg of lutein and 12 mg of zeaxanthin/day showed improved retinal density on multifocal electroretinography and a modest increase in non-edematous foveal thickness (44).

##### 4.1.8.3. Vitamin D

It was observed that plasma concentrations of 25-hydroxyvitamin D ≥75 nmol/L were associated with a reduced probability of developing retinopathy at 3 years (47). Subsequently, in a 2017 retrospective cross-sectional study on over 800 adults emerged that an optimal level of vitamin D is fundamental for reducing the risk and severity of DR (48).

##### 4.1.8.4. Polyphenols

A cross-sectional study by Mayoney examined the effect of flavonoids in diabetic patients who were divided into groups based on the frequency of consumption of fruits and vegetables with high flavonoid contents. It was observed that there was a significant association between a high intake of these foods and lower levels of c-reactive protein (CRP), HgbA1C, and glucose. In addition to lower levels of inflammation and better glycemic control, these patients also had a 30% reduction in the likelihood of DR (49).

##### 4.1.8.5. Vitamin C

It was observed that patients with PDR had a 10-fold lower level of ascorbate in the vitreous humor and a greater tendency to DME (50), and that vitamin C taken with statins decreased NPDR, in a dose-dependent manner, more than statins alone (51). However, regarding the vitamin C–DR relationship, not all studies agree: a Japanese cohort study found that high vitamin C intake (4th quartile) was associated with a 40% reduction in retinopathy risk (34), while two cross-sectional studies showed no association between vitamin C consumption and retinopathy (45, 46), except for an increased likelihood of retinopathy in the 9th decile of vitamin C intake in the study by Mayer-Davis (46). In diabetic subjects, oral supplementation with 1,500 mg of vitamin C reduces capillary endothelial dysfunction (52) and therefore can be a useful support in microvascular pathologies such as RD.

##### 4.1.8.6. B vitamins

Vitamin B1 (thiamine). In addition to controlling intracellular glucose and preventing the activation of the polyol pathway, which is brought on by increased intracellular glucose levels, thiamine is a powerful free radical scavenger (146). This pathway represents one of the mechanisms in the pathogenesis of DR (147). Furthermore, elevated serum thiamine levels protect the vascular endothelium from injury by advanced glycation end products (146, 148, 149). As reported in a 2020 review published in “Eye and Vision” by Shi, for the treatment and prevention of vascular end-organ damage, such as that seen in DR and diabetic nephropathy, high-dose thiamin supplementation (50–100 mg/day) is safe and effective for neuroprotection. Because of the low toxicity, no upper limits (UL) have been recorded (150).

Vitamin B2 (riboflavin). Riboflavin supplementation in humans likely guards against damage caused by oxidative stress, hyperglycemia, and homocysteine (53, 151, 152). Supplementing with vitamins B6 and B12 may also be advantageous since these nutrients lower homocysteine levels (150).

Vitamin B6. A cohort of Japanese 2DM patients was followed for 8 years, monitoring vitamin B6 intake and DR onset. It was noted that low vitamin B6 intake (particularly the lowest quartile of vitamin B6 intake) was correlated with a higher incidence of DR (54). There are various forms of B6, and the naturally occurring active form pyridoxal-5-phosphate (P5P) is the safest and most efficient form for lowering homocysteine levels (153). P5P supplementation may lower the chance of developing DR and diabetes. Vitamin B6 therapy alone, at a dosage of 50–200 mg per day, was associated with a decreased long-term incidence of DR in a small cohort trial of a few participants (150).

Vitamin B9 (folate). The use of supplements with L-methylfolate, B2, B6 (in the form of P5P), and B12 can reduce homocysteine levels, the incidence of DR, and other diabetes-related diseases (150).

Vitamin B12 (Cobalamin). Increased homocysteine levels, as already stated, are linked to decreased cerebral and retinal blood flow, as well as decreased central retinal artery caliber, VEGF expression, and DR (53, 55, 154). Supplementation with vitamin B12 increases the release of nerve growth factor (NGF) and brain-derived neurotrophic factor (BDNF) (155) and allows for the reduction of DR-associated long-term complications (140). The active transport necessary for gastrointestinal absorption of vitamin B12 from food requires the presence of an intrinsic factor, an acidic environment, and an intact intestinal mucosa (156).

##### 4.1.8.7. FA

FA can influence retinopathy through several pathways. First, the accumulation of long-chain FA can lead to activation of the protein kinase C pathway, just as occurs with excess glucose (112, 157). Second, since the retina is an extremely oxidizing and polyunsaturated fatty acids (PUFA)-rich environment, an accumulation of lipids can more easily undergo peroxidation and accumulation of advanced lipoxidation end products (ALEs) (158). Both ALEs and AGEs activate a pro-inflammatory response via the AGE receptor, which activates the proinflammatory transcription factor NF-kB and decreases the antioxidant response (159). Concerning the PUFA and DR relationship in Sazaki's study, an increase in PUFA intake was linked to a lower likelihood of DR occurrence and severity in individuals with well-controlled diabetes, whereas an increase in short-chain fatty acid intake (SFA) was linked to a higher probability of DR occurrence and severity (56). A 500 mg/day consumption of omega-3 FA can greatly lower the possibility of developing DR, as was highlighted in a *post-hoc* analysis of the PREDIMED study by Diáz-López (32). Alpha lipoic acid, an important cofactor of mitochondrial metabolism, has an antioxidant action by counteracting ROS and enhancing the effects of endogenous antioxidants such as glutathione and vitamins C and E (160). The administration of alpha lipoic acid shields the retina's ganglion cells and pigment epithelial cells, in particular, from ischemic damage and apoptosis (161). Furthermore, alpha lipoic acid reduces hyperglycemia and hyperglycemia-induced endothelial dysfunction in type 2 DM patients (57, 58). Daily supplementation with 600 mg of alpha lipoic acid is safe and well-tolerated (150).

##### 4.1.8.8. Zinc

Several chronic disorders, including metabolic syndrome, diabetes-related complications, such as DR, and metabolic syndrome, are known to advance more quickly when there is zinc deficiency. Low serum zinc levels correlate with DM duration, elevated HbA1c levels, hypertension, and microvascular complications. Blood zinc levels gradually decline with DR duration and severity (59).

##### 4.1.8.9. Fiber

A higher risk of getting DR is linked to lower dietary fiber consumption (162). Furthermore, once this complication occurs, intensive glycemic control can slow the rate of development (163). The Mediterranean diet is rich in food sources of fiber, such as fruits, vegetables, and unrefined carbohydrates, and has been associated with a lower incidence of DR (30, 31).

##### 4.1.8.10. Hydratation

Results emerging from the review by Sherwin et al. showed that chronic activation of the renin-angiotensin-aldosterone system (RAAS) may be implicated in the pathogenesis of DR and glaucoma, thus suggesting a possible new therapeutic target on which to base new studies' intervention (105). The cross-sectional population analysis of the 2005–2008 NHANES study (5220 US adults 40 years of age and older) also found that low levels of hydration, as assessed by measured (or calculated) formulas based on blood levels of glucose, sodium, potassium, and urea (limited to subjects aged ≥65 years), were associated with an increased risk of DR (60). So, the intake of adequate quantities of water, equal to 1.5–2 liters per day, is a fundamental objective to achieve.

##### 4.1.8.11. Gut microbiota

Beli (164) first described the link between the gut microbiome and DR in differently fed rodents. The intermittently fasting mice exhibited retinal histology that was comparable to that of non-diabetic controls, while the *ad libitum* diet animals displayed ocular symptoms of DR. Intermittently fasted rodents showed increased Firmicutes to Bacteroidetes ratio and changes in bacterial metabolites, with increased levels of taurochenodeoxycholate (TUDCA) derived from bile acids and known to have anti-inflammatory effects. TUDCA enters the bloodstream and activates GPBAR1, even referred to as TGR5, the TUDCA receptor in the retina. The results imply that intermittent fasting may protect against DR by increasing TUDCA levels and, in addition, TGR5 could represent a novel therapeutic target for the diabetic retina (165). Regarding the role of prebiotics, oligofructose, used alone or in combination with metformin, is effective in preventing the development of DM and its microvascular complications, opening the door for new treatment approaches and research ideas (166). These results suggest that the growth of beneficial bacteria in patients with healthy diets, either through pre- and probiotics, or even through intermittent fasting, could protect against the development of T2DM (167).

##### 4.1.8.12. Physical activity

Increased PA reduced the risk of its onset (61, 62). Higher levels of PA were shown to be independently linked to a decreased incidence of DR in type 2 DM patients (63). A minimum of 30 min of PA, 5 days a week, might minimize the risk of DR advancement by 40% (168). Conversely, it has been revealed that diabetic subjects who lead a sedentary lifestyle have a higher risk of developing DR than those who live actively (169). The results of a meta-analysis also revealed a possible mechanism of impact of PA on DR due to improved glycemic control (170). An alteration in 25-hydroxyvitamin D levels could be another probable mechanism. Supporting evidence is the finding in subjects of all ages that 25-hydroxyvitamin D levels improve with increased PA (64–67, 171). Low levels of 25-hydroxyvitamin D in the blood have been linked to an increased risk of macrovascular and microvascular events, including DR (68). Furthermore, exercise has been shown to modulate oxidative stress (172). Research on animal models has shown that exercise reduces oxidative stress in the retinas of DR mice (173–176). It should be remembered, however, that high-intensity resistance and aerobic exercise should be avoided in diabetic patients with DR to lower the risk of vitreous hemorrhage or retinal detachment (69, 177). Moreover, the risk of vitreous hemorrhage is increased by any exercise which can lead to a rise in systolic blood pressure (178, 179). In animal model studies of diabetic disease, resistance exercise has been shown to lead to increased muscle mass (180). Skeletal muscle is an essential reservoir of glucose in the body, and exercise is a powerful stimulator of glucose uptake, which in part is stored within skeletal muscles (181). Resistance exercise has a direct impact on skeletal muscle and may be used to manage individuals with DM2 (182).

### 4.2. AMD

In the transversal AREDA study conducted on 4,088 participants (whose eyes were divided into three groups: controls, early AMD, and advanced AMD), two major DPs were identified: the Western pattern and the Oriental pattern by using a food frequency questionnaire (FFQ) with subsequent factorial analysis. The first pattern had a higher prevalence of progressive AMD, while an “oriental” dietary style appears to be protective against this pathology (183). Furthermore, according to a recent review, for both early and late AMD, abdominal obesity would be a risk factor (184). In a 2013 cohort study that enrolled 1,760 subjects aged ≥55 years, the authors tried to provide epidemiological evidence for the possible relationship among serum levels of homocysteine, vitamin B12, and folate, and the risk of AMD, finding that high homocysteine levels, as well as a vitamin B12 or folate deficiency, were linked to a higher incidence of AMD at age 10 years; this risk was decreased by 47% with vitamin B12 supplementation (55). Moreover, the effects of several dietary sources, including omega-3 and omega-6 fatty acids, mono-, polyunsaturated, and saturated fats, total fats, trans fats, and cholesterol, on the risk of AMD have been investigated. In particular, omega-3 has anti-inflammatory properties and, when transformed into neuroprotectin, can help prevent oxidation-induced apoptosis in retinal cells and support the fluidity of the photoreceptor membrane (185). The polyunsaturated fatty acids EPA and DHA are linked to a lower incidence of AMD and play a preventive role in the course of the illness, according to a 2018 review of epidemiological, clinical, and experimental data. Indeed, in humans, the retina has a lipid profile that is especially high in long and very long-chain polyunsaturated FA, which is crucial for maintaining retinal structure and function (184). In the US Twin Study of AMD, a cross-sectional study performed on 681 twins, of which 222 subjects had intermediate or advanced stage AMD and 459 did not or just exhibited initial signs of the disease, it was demonstrated that a higher omega-3 FA level (upper quartile, corresponding to a mean daily intake of 0.35 g of omega-3, vs. lower quartile, corresponding to 0.06 g/day) was inversely related to AMD, with a significant risk reduction observed primarily in subjects with a lower than average intake of linoleic acid (an omega-6 FA) (1.8 g/day) (79). In the Blue Mountains Eye Study, 2,335 participants aged 49 years and older underwent reevaluation at 5 years for the development of AMD. Results showed that those in the highest quintile of omega-3 fatty acid intake (0.52–2.11%, expressed as a percentage of total energy intake) had a lower risk of early AMD onset than those in the lowest quintile (0.05–0.26%), with a 40% reduction in incidence when consuming fish at least once a week (80). Consumption of 1–2 portions of nuts per week (compared to less than one portion per week) was also related to a lower risk of early AMD onset, with a protective impact in comparisons of retinal pigment abnormalities reported in non-smokers, subjects with a lower-than-average total cholesterol-to-HDL-cholesterol blood ratio, and those with higher-than-average beta-carotene intake (6836 g/day) (81). Within the Age-Related Eye Disease Study (AREDS), 4,519 subjects (60–80 years) provided an estimate of habitual nutrient intake through self-administered, semi-quantitative FFQ, from which the study showed that those in the top quintile of total long-chain-omega-3 intake (0.110% of total energy intake) and DHA (0.061%) had a lower risk of neovascular (NV) AMD (NV AMD) than bottom quintile (0.013% for total omega-3 and 0.010% for DHA). In general, higher consumption of fish was inversely related to NV AMD, while arachidonic acid taken with food was directly associated with the incidence of this pathology (82). Several other studies have looked into the connection between lipid intake and the risk of AMD, including a cohort study with 6,734 people (aged 58 to 69) who completed the FFQ and also reported using supplements (ascorbic acid, vitamin E, cod liver oil, and fish oil). A greater trans-fat intake was linked to a higher prevalence of late AMD, whereas higher omega-3 FA and olive oil intake would lessen the incidence of both early and late AMD, respectively (upper quartile, 1.4 g/d vs. lower quartile, 1.0 g/d; OR, 0.85; 95% confidence range, 0.71–1.02; *P* = 0.03). However, neither monounsaturated FA nor oleic acid, of which olive oil is particularly rich, were associated with late AMD; presumably, therefore, other non-FA contained in this oil could be responsible for its protective effect. Conversely, conflicting results emerged from an Australian study on 254 subjects diagnosed with early AMD, in which the possible progression of the disease at 7 years was evaluated: these findings contribute to a relationship between omega-3 intake (as measured by FFQ) and the development of AMD, potentially demonstrating how excessive consumption of a drug having therapeutic effects can be hazardous (83). Furthermore, high consumption of total, saturated, and monounsaturated fats was linked to an elevated risk of age-related maculopathy in the POLANUT trial, which involved a sample of 832 people from southern France. While no significant correlation emerged with polyunsaturated FA intake, a 60% decrease in the risk of maculopathy was associated with fatty fish consumption frequency (more than once per month vs. less than once per month) (84). The multi-center, case–control study by Seddo examined 504 controls without AMD but with other ocular pathologies, as well as 349 patients (55–80 years) with advanced neovascular AMD. It concluded that higher consumption of certain types of fats, especially mono- and polyunsaturated-FA of vegetable origin, may be linked to an increased risk of advanced AMD, while diets high in omega-3s and fish (two or more servings/week vs. less than one serving/week) seemed to be inversely associated with this risk, but limited to subjects with low linoleic acid intake ( ≤ 5.5 g vs. ≥5.6 g) (85). Given that the GI of foods appears to play a role in the pathogenesis of AMD, a significant group of studies have looked into the potential involvement of carbs in AMD. Low dietary GI values (dGI75.2 vs. 81.5, computed as the average of GI of specific items weighed by the presence of carbs) were linked to a lower chance of developing advanced forms of AMD, according to an analysis of the data from the AREDS study: More specifically, it was discovered that a dGI reduction of 6 units (roughly equivalent to substituting 5 slices of white bread with 5 slices of whole grain bread in a subject's daily diet who consumes 250 g/day of total available carbohydrates) could prevent 8% of advanced AMD cases for 5 years. The production of advanced glycosylation products, the aggregation and precipitation of glycosylated protein aggregates, and the ensuing inflammatory and angiogenic responses have all been linked to higher post-prandial glycoxidative stress caused by high GI foods. Furthermore, the compensatory hyperlipidemia that occurs in the late post-prandial phase following the intake of high GI foods could also play a role in the pathogenesis of AMD (86). A higher mean dietary GI (lower quartile vs. upper quartile) is associated with a higher 10-year risk of developing early AMD, according to the Australian Blue Mountain Eye Study (3,654 participants, 49 years and older, examined at baseline in 1992–1994, of whom 2,335 were re-examined after 5 years, and 1952 after 10 years). This is after adjusting data for potential confounders and diet constituents. On the contrary, a greater consumption of whole-meal bread and cereals (in particular, those with a lower GI) was related to a reduction in this risk. In 1993, the Eye Disease Case-Control Study Group found that participants with intermediate and high blood levels of carotenoids had a much lower chance of developing neovascular AMD than those with low levels—equivalent to half and one-third, respectively. Within the same study, surveys performed on a sample of 356 patients with advanced-stage AMD (55–80 years) and 520 controls showed that subjects in the top quintile of dietary carotenoid intake had a risk of AMD 43% lower than subjects in the bottom quintile, and how, among the specific carotenoids, lutein and zeaxanthin (mainly found in green leafy vegetables) had the strongest association with a reduced risk of AMD (consuming spinach and collard greens more frequently was linked to a significantly decreased incidence of AMD) (87). A case–control study on the intake of antioxidants (72 patients and 66 controls) revealed that AMD was almost two times as common in patients who consumed fewer antioxidants and lutein than the typical person compared to those who consumed more, indicating a clear dose–response relationship (88). Vitamin A, which the body stores as retinol, is the source of several carotenoids. Even after adjusting for variables, demographics, and specialists, the National Health and Nutrition Examination Survey (NHANES) study found a negative correlation between the frequency of consumption of vitamin A-rich fruits and vegetables and the prevalence of macular degeneration in subjects under the age of 45 years (27). These observational studies collectively imply that lutein and zeaxanthin are the carotenoids that benefit the retina the most out of all those under investigation. These effects seem to be exclusive to certain types or stages of macular degeneration, with advanced disease benefiting most from a lower risk of damage. It is reasonable to speculate that vitamin C's potent antioxidant activities may be crucial in the onset and progression of the illness given the significance of oxidative stress on the etiopathogenesis of AMD. Most of the early studies were case–control studies. In 2002, Simonelli et al. analyzed the oxidative status of the serum/plasma in 48 Italian patients with macular degeneration (19 with the early form and 29 with the late form) and 46 healthy subjects, showing that subjects with late pathology had plasma levels of vitamin C, vitamin E, total carotenoids, and beta-cryptoxanthin compared to patients with early AMD, but with no differences in plasma levels of vitamin C between patients with ocular disease and healthy controls (89). Other observational studies have confirmed a small effect of vitamin C on the risk of macular degeneration. Data obtained from 4,519 participants in the AREDS study, which suggested a reduced probability of developing neovascular AMD in subjects with the highest vitamin C intake, were then not confirmed following the addition of covariates (82). Even the multicenter Eye Disease Case–Control Study (EDCCS), which included 520 controls with other eye diseases and 356 patients with advanced-stage AMD (55–80 years), failed to detect any statistically significant link between vitamin C consumption and risk of AMD, even though the data appeared to point to a lower risk among those with the highest intake of vitamin C (particularly that contained in food) (87). Following multivariate adjustment, the examination of NHANES data from 1971 to 1972 revealed that there was no correlation between vitamin C intake and the prevalence of AMD at any stage (27). In addition to serving as a catalyst for more than 50 different enzymes, zinc also controls the expression of genes and contributes to the structure of proteins, making it a vital component of many physiological processes (186). Furthermore, zinc, together with copper, is an essential microelement for the retina, particularly concentrated in photoreceptors and pigmented epithelium of the human eye. Zinc and copper also act as cofactors for numerous ocular enzymes, including superoxide dismutase, a component of the main antioxidant system that modulates oxidative stress in the body. Oxidative stress and a reduced antioxidant capacity have been included among the possible pathogenetic factors implicated in the genesis of AMD, as the retina, and in particular the RPE, are particularly susceptible to oxidative stress due to high oxygen tension, high content of polyunsaturated fats, and intense exposure to light. These factors have led some researchers to hypothesize that taking zinc supplements may benefit retinal health (187). Zinc was a component of the antioxidant mixture given to the intervention group in the AREDS study. Participants were first randomized into four groups at random and given one of the following treatments per day: (1) antioxidants (vitamin C, 500 mg; vitamin E, 400 IU; and beta-carotene, 15 mg); (2) zinc, 80 mg; and copper, 2 mg, as cupric oxide; (3) antioxidants plus zinc; and (4) placebos. Data on subjects who took zinc (thus including both those who took zinc alone and those who took zinc plus antioxidants) proved to be suggestive of a reduction in the risk of developing advanced forms of AMD, while no significant effect emerged in subjects taking antioxidants (including both the antioxidants-only group and the antioxidants-plus-zinc group). A statistically significant risk reduction was seen for antioxidants + zinc and suggestive for zinc alone, but not for antioxidants alone, when individual intervention groups were compared with placebo. Additionally, considering only individuals with the most severe forms of AMD, the size of the risk decrease increased (90). The Beaver Dam Eye Study, a prospective population-based study that initially enrolled 4,926 participants in 1990 and then reexamined 3,722, 2,962, and 2,375 participants in 1993–1995, 1998–2000, and 2003–2005, respectively, has revealed a higher risk of late AMD in users of supplements based on vitamins A, C, E, and zinc (91). In more recent times, attempts have been made to analyze the associations between illness and diet not so much understood as a single nutrient or food, but as a food style, comparing healthy styles and not starting from the large studies done in the past. The first and most important investigation of the Mediterranean diet and AMD was the French prospective cohort study by Merle et al. from 2015, conducted on 2,525 participants of the AREDS study (in which 1,028 eyes were found to have progressed to an advanced form of AMD for 13 years). The alternate Mediterranean Diet score (aMeDi, range: 0–9, from non-adherent to fully adherent) was calculated for each subject using a validated, self-administered, semi-quantitative FFQ. This score is widely used to assess adherence to the Mediterranean Diet in the US population based on the individual intake of nine components: vegetables, fruit, legumes, whole grains, nuts, fish, red and processed meats, alcohol, and the ratio of monounsaturated and saturated fats. In addition, 10 genetic loci associated with AMD located in seven different genes were determined and analyzed as covariates (for inherited predisposition). A high aMeDi score (6–9) was significantly associated with a 26% reduced risk of progression to advanced disease after adjusting for demographic, behavioral, ocular, and genetic covariates (HR: 0.74; 95% CI: 0.61–0.91; *P*-trend = 0.007). Furthermore, the aMeDi score appeared to be associated with a lower risk of incidence of advanced disease among subjects carrying non-at-risk alleles, while no association with AMD emerged among subjects homozygous for the risk allele. Greater adherence to the Mediterranean diet, therefore, appears to be associated with a reduced risk of progression to advanced disease, a risk that can be modified by genetic susceptibility. Finally, the data collected demonstrated that two components of the aMeDi score, in particular, the consumption of fish and that of vegetables, were associated with a lower risk of progression (92). Surveys conducted on 4,202 participants in the Rotterdam Study, through the administration of a validated FFQ comprising 170 items and classifying the data obtained on dietary intakes in nine food patterns according to the Health Councils guidelines, showed an association of fish with 24% reduced risk of AMD occurrence (mean follow-up of 9.1 ± 5.8 years), while no other association with single food categories reached statistical significance. Furthermore, the authors highlighted that only one DP, the one characterized by the intake of ≥200 g/day of vegetables, ≥200 g/day (two servings a day) of fruit, and ≥32 g/day (equivalent to two servings per week) of fish, was significantly associated with a lower risk of developing AMD (hazard ratio 0.58 [95% confidence interval 0.36–0.93]) (93). An additional survey conducted on 4,088 subjects participating in the AREDS study identified, based on the data obtained through FFQ, two major DPs (Oriental and Western) and eight minor DPs (subgroups or extensions of one of the two main patterns, generally including a smaller number of characterizing foods). The two major patterns were significantly associated with both early (OR Oriental pattern: 0.74; OR Western pattern:1.56) and advanced AMD (OR Oriental pattern:0.38; OR Western pattern:3.70), while no minor pattern showed a correlation with early AMD, and only four of these were found to be significantly associated with advanced AMD, including Steak pattern [similar to the Western DP; OR comparing the highest to the lowest quintile of the pattern score = 1.73 (95% confidence interval: 1.24–2.41; *P* trend = 0.02)], Breakfast pattern [cereals, fruit, and fruit juices; 0.60 (0.44–0.82); *P* trend = 0.004], Caribbean pattern [white meat, fish, rice, low-fat dairy, and offal; 0.64 (0.47–0.89; *P* trend = 0.009)], and Peanut pattern [peanuts, snacks, high-fat dairy, and sweets; 0.64 (0.46–0.89; *P* trend = 0.03)]. The data collected suggested that specific foods may harbor potentially beneficial effects (peanuts, pizza, coffee, and tea) or harmful effects (salad dressing) against the development of AMD (94). Amirul Islam discovered six food patterns (or factors) that are characterized by a preponderance of consumption of fruit (Factor 1), vegetables (Factor 2), grains, fish, steamed or boiled chicken, vegetables, nuts (Factor 3), red meat (Factor 4), processed foods, such as cakes, cookies, pastries, and desserts (Factor 5), and salads (Factor 6). Patterns from factors 1–3 were associated with a lower prevalence of AMD, while factors 4 and 6 were associated with a higher prevalence of advanced AMD. Notably, factor 4, which also included processed fish, eggs, and a low intake of whole grain foods (wheat or rye bread) was associated with an increased risk of late AMD, but not early AMD (OR = 1.46; 95% CI:1.0–2.17). The typical Western DP containing mostly processed foods (Factor 5) was found to have no significant association with AMD. In contrast, the latter pattern also included foods such as dairy, tea, and peanuts, which are known to protect against AMD, demonstrating that the impacts of potentially harmful foods featured in the DP may be mitigated by the consumption of beneficial foods (95).

#### 4.2.1. Physical activity

An active lifestyle, defined by at least 3 h of daily low-to-moderate intensity physical activity, is related to a decreased risk of AMD, according to a recent meta-analysis of nine cross-sectional studies that assessed the effects of PA on AMD in 15 research, with a protective association against both early AMD [8 studies, *n* = 38,112, odds ratio (OR) 0.92, 95% confidence interval (CI) 0.86–0.98] and late AMD [7 studies, *N*=28,854, OR 0.59, 95% CI 0.49–0.72] (188).

### 4.3. Cataracts

The Women's Health Study (WHS) is the largest prospective cataract study that also correlates total fruit and vegetable consumption (96). The study boasts an average of 10 years of follow-up, in which there were 2,067 cases of cataract onset and 1,315 cases of lens replacement due to cataracts. Compared with women in the lowest fruit and vegetable consumption quintile, women in quintiles 2–5 (≥3.4 servings/day) had a moderate (10–15%) reduction in the risk of cataracts (*P* = 0.05). In the 2013 study by M. Pator-Valero, an inverse association between increasing quartiles of fruit and vegetable intake and the prevalence of cataracts was demonstrated. The study's stated consumption was much higher than what other studies had described. The WHO recommendation of five or more servings of fruit and/or vegetables per day (>400 g/day), with a median of 440 g/day, was actually met by 50% of the Spanish study population (IQR 226). The Alicante diet (study population) is a Mediterranean diet abundant in fruits and vegetables, particularly citrus fruits, and offers high levels of antioxidant vitamins (99) compared to the best American diets of other studies examined. Among the antioxidants examined in the Spanish study, dietary vitamin C has a more consistent effect on cataract prevalence. The results show that daily intake of vitamin C in the diet >107 mg/day are inversely linked with a decreased risk of developing cataracts (*P* trend between the four quartiles = 0.047). Compared with the lowest quartiles, with vitamin C intakes between 13 mg/day and 83 mg/day, vitamin C intakes between 83 and 107 mg/day were discovered to be 38 times less likely to be related to cataract prevalence and intakes between 107 and 143 mg/day were associated with a 51% lower probability of cataract development. Arrives at 54% with intakes between 143 and 408 mg/day. These data are consistent with previous studies that demonstrated that human eye tissues become saturated with vitamin C with dietary intakes between 200 and 300 mg/day (189). An analysis of the Nutrition and Vision Project (97) also obtained similar results observing a significant 48% reduction in the likelihood of nuclear opacity for vitamin C intakes between 140 and 180 mg/day, a reduction of 53% for intakes between 180 and 240 mg/day, and of 66% for intakes between 240 and 360 mg/day compared to the intakes of the highest quintiles (<140 mg/day). The French study POLA (84) instead found an inverse association between nuclear cataracts and plasmatic zeaxanthin [OR = 0.23 (0.08–0.68)], thus concluding that xanthophylls are important for the prevention of ocular compared to individuals who had low plasma zeaxanthin levels (0.04 mol/L). Nuclear cataract risk was reduced by 75% in people with high plasma zeaxanthin levels (>0.08 mol/L), but not for other types of cataracts. The authors found no association between lutein and cataracts of any type. The CAREDES study (98), composed of women previously enrolled in an observational study and who were above and below the 78th and 28th percentiles, respectively, for consumption of lutein and zeaxanthin, demonstrated that women whose overall scores for HEI-95 (Healthy Eating Index-95) were in the highest vs. lowest quintiles had diets that were less rich in fat, saturated fat, in particular, and contained less sodium. The prevalence of cataracts was related to low values for most of the subscale scores (vegetables, fruit, milk, cereals, total saturated fat, and food variety in general). Furthermore, this study shows that meat consumption is directly related to cataracts (*p* = 0.07). The analysis of sodium and cholesterol consumption did not lead to any specific results. Two studies on the same population in Iran (100, 101) highlighted how DPs rich in sodium and trans-fats were linked to a higher prevalence of cataracts. Ghanavati used a case–control study evaluating the association of cataracts with a healthy eating style, the Healthy Eating Index (HEI). The analysis of FFQ led to dividing the population into three sub-groups with respect to the diet followed. The two categories of HEI were found to be protective against cataracts, while the population in the lowest quartile [OR = 0.19 (95% CI: 0.09–0.4); *P* < 0.01] had the greatest prevalence. Factor analysis was used on dietary data (101) to extract nutritional patterns and identified two particularly inadequate nutritional patterns, defined as sodium regimen and fatty acid regimen. Sedaghat has redivided the nutritional models into five models based on nutrients. The regimens are as follows: (1) sodium regimen: included niacin, thiamine, high amounts of carbohydrates and proteins, zinc, vitamin B6, and sodium; (2) fatty acid regimen featuring oleic acid, monounsaturated fatty acids (MUFA), PUFA, linoleic acid, trans FA, vitamin E, and saturated fat; (3) mixed regimen represented a high intake of vitamin B12, vitamin D, cholesterol, and calcium; (4) the antioxidant regimen had high intakes of beta and alpha carotene, vitamin A, and vitamin C; and (5) omega-3 regimen contained a high intake of DHA and EPA. In the crude, multivariate analysis, the sodium model was associated with an increased risk of cataracts (OR = 1.97, 95% CI: 1.09–3.96). The FA pattern (this model represents a surrogate for meats and processed foods) was associated with high risk (OR = 1.94, 95%CI: 1.1–3.86), while the antioxidant regimen was associated with 79% reduced risk compared to the sodium regimen. Finally, the omega-3 model was negatively associated with cataract risk (*P* = 0.04). The narrative review by Chong in 2008 suggests that the risk of cataracts can be reduced by adhering to diets high in vitamin C, xanthophylls (lutein and zeaxanthin, present not only in the macula but also in the lens), omega-3 FA, and avoiding frequent and abundant intakes of simple carbohydrates with a high GI (103).

#### 4.3.1. Hydration

The high-water content in the eye, as well as the peculiar fluid regulation system in its context, suggest that the state of hydration may also play an important role in determining the health or disease state of the eye itself (105). A 2015 review suggested that dehydration correlates with the onset of some eye diseases, such as dry eye syndrome, cataracts, retinal vascular diseases, and refractive defects (105). In particular, the cornea, the main refractive medium of the eye, is made up of ~80% water, and its transparency mainly depends on its state of hydration. Indeed, changes in the state of hydration of the cornea can result in a change in its central thickness and the ability to recover from such changes decreases with age (190, 191). This could also affect the outcome of cataract surgery (105). Indeed, diabetes has also been observed to increase the risk of developing cataracts, as well as in diabetic patients suffering from cataracts, the total water content of the eye's lens system is reduced (192, 193). In a case–control study conducted in India in 1989 on 434 cases and 930 controls (30–69 years), 38% of the cases suggested that the cause could be attributed to episodes of severe dehydration, in a dose-dependent manner (102). Given this background, water must therefore be taken in a quantity of 1.5–2 liters per day, as per the indications of all international guidelines for a healthy diet.

#### 4.3.2. Physical activity

A recent review (104) evaluated the outcome of PA on cataracts, finding that regular activity decreases the rate of progression and risk of incidence. Results from prospective cohort studies accessible and examined in this review revealed that greater PA was inversely related to cataract risk and that the association was significant in studies that measured the metabolic equivalent of task (MET) PA as opposed to studies that measured it as a weekly activity. According to a dose–response analysis, each increase of 6 METs/day resulted in a 2% reduction in the chance of developing cataracts. The ocular lens is highly susceptible to oxidative damage as it is rich in polyunsaturated FA and the presence of greater quantities of ROS has great toxicity on the components of the lens itself, such as the crystalline proteins, whose damage leads to the development of opacities (194, 195). From this point of view, PA could reduce the levels of oxidative stress by increasing the activity of antioxidant enzymes and thus favoring the prevention of cataracts.

## 5. Conclusion

Many common eye diseases, in particular DR, AMD, and cataracts, are treatable and preventable, especially in the first phase in which they occur. Lifestyle, especially nutrition and physical activity, plays an essential role. To create a food pyramid that makes it simple for people who are at risk of developing DR, AMD, and cataracts to decide what to eat, this narrative review analyzed the most recent research on the best dietary strategy needed to avoid the development of these pathologies. In preventive terms, the subjects who can benefit most from following the indications given in the pyramid are the following: diabetic and hypertensive subjects as they are at greater risk of diabetic retinopathy since both pathologies tend to damage the retina; subjects who are hypertensive and smokers as they are at increased risk of age-related macular degeneration; subjects suffering from other eye diseases such as glaucoma or uveitis, diabetics, and who have undergone prolonged therapies with cortisone as they are at greater risk of diabetic retinopathy. The pyramid illustrates the recommended daily diet: three portions of grains with low GI (for high fiber and zinc content), five portions (**≥**200 g/die) of fruits and vegetables, especially spinach and broccoli and cooked zucchini and green leafy vegetables, orange, kiwi, grapefruit (for luteina/zeaxantina at least ≥942 μg/die content, are to be preferred), light yogurt (125 ml), skim milk (200 ml), EVO oil (almost 20 mg/day for high vitamin E and polyphenols content), and nuts or oilseeds (20–30 g/day, for zinc content, at least **≥**15.8 mg/die); and weekly: fish (4 portions, for omega-3 content, EPA+DHA at least 0.35 as far as 1.4 g/day), white meat (3 portions for vitamin B12 and folic acid content), legumes (2 portions for vegetal proteins), eggs (2 portions for lutein/zeaxanthin content), fresh and low-fat cheeses for the content of vitamins of group B), and red or processed meats (once/week) and microgreen (at least once a week). There are two pennants at the top of the pyramid: one green indicates the need for individualized supplementation (if daily requirements cannot be met through diet, omega-3 supplementation and L-metilfolate may be a useful strategy with a great benefit-to-cost ratio) and one red indicates the presence of certain foods that are prohibited) (salt and sugar). Finally, 30 to 40 min of aerobic and resistance workouts must be done three to four times per week, and the intake of adequate quantities of water, equal to 1.5–2 liters/day, is a fundamental objective to achieve. Another important topic on which most of the literature agrees is the importance of maintaining a BMI between 19 and 25 kg/m^2^. Finally, in these conclusions, it is necessary to remember a topic that will be the subject of many studies in the near future: the relationship between intestinal microbiota and eye diseases because the microbiota can influence several metabolic pathways involved in the regulation of ocular health. Inflammation and hyperglycemia can lead to intestinal permeability of microbial products, which can in turn bind to ocular receptors and transmit inflammatory signals. The gut microbiota influences bacterial and host-derived metabolites, which signal distally to the brain and eye and influences systemic lipid metabolism, and has been shown to influence the lipid composition of the retina.

## Data availability statement

The original contributions presented in the study are included in the article/supplementary material, further inquiries can be directed to the corresponding author.

## Author contributions

MR, SP, and AR contributed to the conception and design of the study. AC, CR, and AT wrote the first draft of the manuscript. CG, GB, and GP wrote sections of the manuscript. All authors contributed to the manuscript revision, read, and approved the submitted version.
